# The influence of social network on depressive and anxiety symptoms during the COVID-19 pandemic: findings from a Swedish cohort study

**DOI:** 10.1192/bjo.2025.10915

**Published:** 2025-12-23

**Authors:** Gillian L. Murphy, Emily E. Joyce, Anikó Lovik, Elísabet U. Gísladóttir, Katalin Vincze, Anna K. Kähler, Emma M. Frans, Unnur A. Valdimarsdóttir, Patrick F. Sullivan, Mary Barker, Fang Fang

**Affiliations:** Institute of Environmental Medicine, Karolinska Institute, Stockholm, Sweden; Institute of Psychology, Leiden University, The Netherlands; Department of Medical Epidemiology and Biostatistics, Karolinska Institute, Stockholm, Sweden; Centre of Public Health Sciences, Faculty of Medicine, School of Health Sciences, University of Iceland, Reykjavik, Iceland; Department of Epidemiology, Harvard TH Chan School of Public Health, Boston, Massachusetts, USA; Departments of Genetics and Psychiatry, University of North Carolina, Chapel Hill, North Caroline, USA

**Keywords:** Social network, depression, anxiety, COVID-19, cohort study

## Abstract

**Background:**

The COVID-19 pandemic disrupted daily social interactions, potentially affecting mental health. Understanding the risk of depressive and anxiety symptoms is essential for guiding mental health strategies during future crises.

**Aims:**

To explore how social networks influenced mental health outcomes during the pandemic and how these relationships changed over time.

**Method:**

Data from the Omtanke2020 study, a prospective cohort study of Swedish adults, were analysed using structural equation modelling (*N* = 10 918). Surveys at baseline and follow-up at 6 and 12 months assessed social networks, including structural components (e.g. relationship status, frequency of social contact) and perceived components (e.g. emotional support from family, feeling safe at home). Cross-lagged panel modelling was used to observe changes over time in the associations between social network indicators and depressive and anxiety symptoms.

**Results:**

Stronger perceived social support – specifically closeness to family, perceived warmth or love from others and increased societal cohesion – were negatively correlated with depressive and anxiety symptoms across all time points (*β* coefficients = −0.14 to −0.23, all *P* < 0.001). Social network variables consistently predicted mental health outcomes, with effect sizes remaining relatively stable over time (*β* coefficient = −0.17 at baseline, *β* coefficient = −0.21 at 1-year follow-up).

**Conclusions:**

This study highlights the protective role of the social network – namely perceived social support – in combatting depressive and anxiety symptoms during the COVID-19 pandemic. Interventions that strengthen close interpersonal ties and community cohesion may help mitigate mental health impacts during future public health crises.

Social networks are complex systems of interpersonal relationships that have long been acknowledged to be highly influential on mental health and general well-being.^1,2^ Strong connections with friends, family and community members enhance psychological resilience, offering belonging, emotional reinforcement and practical aid.^3,4^ Robust social networks are consistently associated with lower rates of depression and anxiety, particularly during stressful periods.^5^ The 2019 coronavirus disease (COVID-19) pandemic is a reminder of the importance of social relationships while highlighting their complex interplay with mental health. Physical distancing measures aimed at curbing viral spread drastically altered daily life, fostering isolation and uncertainty.^6,7^ These disruptions transformed positive social interactions into potential risks for viral transmission, yet perceived social support remained a vital buffer against psychological distress.^8^

While studies have examined the mental health impacts of social networks during the pandemic, many relied on isolated time points, small samples or specific subgroups such as adolescents or healthcare workers.^9–12^ Comprehensive investigations across diverse populations and time points are needed to assess the broader implications of social networks on mental health during crises. Sweden’s unique pandemic strategy, which emphasised voluntary guidelines over mandatory lockdowns, provides a valuable context for exploring these dynamics.^13,14^ This study investigates the relationships between social networks and depressive and anxiety symptoms over 12 months in a large Swedish cohort, addressing gaps in previous research. We aimed to examine (a) whether social network characteristics are associated with symptoms of depression and anxiety at each time point, (b) how these associations changed over the course of the pandemic and (c) whether there are lagged, bidirectional effects between social network characteristics and mental health over time.

## Method

### Study population and data source

We used data from the Omtanke2020 study, a prospective cohort study established in June 2020 to investigate the mental health of Swedish adults during the COVID-19 pandemic.^15,16^ Participants were eligible if they were ≥18 years old, Swedish speaking and in possession of a Swedish personal identity number and electronic identification. Recruitment occurred from 9 June 2020 until 8 June 2021. Participants either volunteered vi social media campaigns or were actively recruited through existing cohorts at Karolinska Institute – namely the Karolinska Mammography Project for Risk Prediction of Breast Cancer, the Swedish Twin Registry and LifeGene.^15^ Comprehensive online surveys were administered at baseline, every month after recruitment during the first year and annually during the 3 years following recruitment. At baseline, a total of 29 521 adults provided informed consent, with the final baseline cohort consisting of 28 302 participants who answered at least 1 question. Participants were included in the present study if they participated in the baseline and 6- and 12-month follow-up questionnaires, and had non-missing data for the social network questions and measures of depressive and anxiety symptoms.

### Social network

The social network domain comprised both objective and subjective measurements. Objective measurements included: relationship status (married; cohabitating or other firm relationship; single or divorced; widowed), parenthood (yes/no), presence of child(ren) at home (yes/no); and four frequency-of-contact items asking how often participants in the past 2 weeks had spoken on the phone, contacted others via social media, met others in person and left their home. Subjective measurements were seven items asking agreement with statements about emotional support from family, having a specific person to turn to, feeling safe at home, increased family closeness, increased warmth or love from others, perceived increase in societal cohesion and perceived helpfulness to others. Response formats are shown in Supplementary Table 1 available at https://doi.org/10.1192/bjo.2025.10915: contact frequency items had six ordered response categories (several times per day → more than once per week); and perceived items used a five-point Likert scale (do not agree at all → completely agree). All social network items used in structural equation modelling (SEM) analyses were standardised to *z*-scores using cluster resampling by calendar month, to reduce bias from seasonal and calendar-period differences in response distributions over the recruitment period.

### Mental health

#### Depressive symptoms

Depressive symptoms were measured with the Patient Health Questionnaire (PHQ-9), a validated self-report questionnaire used to assess the presence of depressive symptoms.^17^ This questionnaire includes nine items corresponding to each of the DSM-IV criteria for depressive symptoms. Response options relate to how frequently participants had experienced each item during the past 2 weeks, measured using a 4-point Likert scale from 0 (‘not at all’) to 3 (‘nearly every day’). Total scores range from 0 to 27, with a score of 10 or above indicating the presence of moderate to severe depressive symptoms. This measurement tool has consistently shown high sensitivity and specificity for major depression, and is thus a good indicator for clinical diagnosis.^17^ The internal consistency for PHQ-9, based on the Omtanke2020 study at baseline, was *α* = 0.88.^15^

#### Anxiety symptoms

Anxiety symptoms were measured using the General Anxiety Disorder (GAD-7) scale, a validated self-report questionnaire used to assess the presence of generalised anxiety disorder (GAD) symptoms during the past 2 weeks, represented by 7 items that are measured using a 4-point Likert scale. Total scores range from 0 to 21, with a score of 10 or above indicating the presence of moderate to severe anxiety symptoms. Like PHQ-9, GAD-7 is a reliable measurement tool for detecting the presence of GAD.^18^ The internal consistency of GAD-7, based on the Omtanke2020 study at baseline, was *α* = 0.90.^15^

### Covariates

We selected covariates *a priori* based on theoretical and empirical links with both social network indicators and mental health outcomes. Covariates measured at baseline include age (continuous); gender (male or female); current smoking status (yes or no); history of any mental illness (yes or no); recruitment type (via prior cohort participation or social media); body mass index (BMI; <25, 25–30 or >30); problematic alcohol consumption during the past 2 weeks (measured using the CAGE Substance Abuse Screening Tool, where higher CAGE scores indicate alcohol abuse problems and a score of ≥2 is considered clinically significant^19^ (0, 1 or ≥2)); frequency of physical activity during the past 7 days (every day, 4–6 days, 1-3 days or none); and number of chronic diseases (0, 1, 2 or ≥3). We also included several time-varying covariates using data collected at baseline and at 6- and 12-month follow-ups, namely employment status (full-time work, part-time work, not working, retired or student); change in employment (no change, not relevant (i.e. student, retired), lost job or other negative change, positive change, other); economic difficulties (a 5-point scale ranging from ‘very easy to ‘very difficult’ to make ends meet financially in the past 6 months); COVID-19-related worries (a 5-point scale ranging from ‘very worried’ to ‘not at all worried’ about becoming infected with the COVID-19 virus); previous positive COVID-19 polymerase chain reaction test (yes or no); and survey completion date for baseline and 6- and 12-month follow-up. For subgroup analysis only, age was categorised into three groups: 18–39 years, 40–59 years and ≥60 years.

### Statistical analysis

Missing data for the PHQ-9 and GAD-7 scales were handled using imputation methods based on the joint distribution.^15^ For the entire study population, means (± standard deviation), medians (interquartile range) and frequencies (percentages) were calculated, where appropriate. Descriptive statistics are presented for males and females separately. Missing covariate data were included in all analyses, with missing answers treated as a separate category. Statistical analyses were performed using Stata/BE 18.0 for Windows (StataCorp LLC, College Station, Texas, USA; https://www.stata.com) and the lavaan package in R version 4.3.1 for Windows (The R Foundation, Vienna, Austria; https://cran.r-project.org).

#### SEM

Structural equation modelling was employed to examine the correlations of social networks with depressive and anxiety symptoms at each time point. Because social network might have a delayed influence on mental health outcomes, we employed cross-lagged panel modelling (CLPM) to explore the potentially lagged effects of social network on depressive and anxiety symptoms across the three waves. CLPM is a conventional SEM approach that estimates both autoregressive effects and cross-lagged paths, thereby allowing assessment of influences between variables over time. The hypothesised cross-sectional and cross-lagged models are presented in Supplementary Figs 1 and 2, respectively.

Cross-sectional and cross-lagged models were first analysed without covariate adjustment. In the multivariable analysis, a two-step approach was used. First, all social network and mental health variables were individually regressed on the aforementioned covariates. The residuals obtained from these regression models were used to generate new analysis variables, which were subsequently incorporated into the models to adjust for covariates. For baseline data, three models were used: (a) adjusted for age and gender; (b) additionally adjusted for history of mental illness, chronic diseases, BMI, smoking status, CAGE score, physical activity and recruitment type; and (c) additionally adjusted for previous COVID-19 infection, COVID-19-related worries, economic difficulties, employment status and response period. For 6- and 12-month follow-ups, we adjusted for previous COVID-19 infection, COVID-19-related worries, economic difficulties, employment status, changes in employment and response period. Variables from adjusted models were then fit cross-sectionally and cross-lagged to ascertain the relationships between social network components and anxiety symptoms and depressive symptoms at the three time points.

Model parameters and standard errors were estimated using maximum likelihood. Model fit was assessed using the *χ* ^2^ goodness of fit statistic, the comparative fit index (CFI), the Tucker–Lewis index (TLI), the standardised root mean squared residual (SRMR) and the root mean square error of approximation (RMSEA). A good fit indicates that the model accurately represents the relationships among the variables. A good model fit was defined as CFI ≥ 0.95, TLI ≥ 0.95, SRMR ≤ 0.05 and RMSEA ≤ 0.05.^20^ The Akaike information criterion (AIC) was also used to compare different models, with a lower AIC value indicating a better model fit.

The analytic procedure started with the hypothesised models containing all social network variables (Supplementary Figs 1 and 2). However, initial model fit did not meet the established criteria for good fit. To improve model fit while maintaining theoretical integrity, the trimming procedure outlined by Meyers et al^21^ was applied. This involved systematic removal of non-significant paths or variables from the model to improve its fit and parsimony, reassessing model fit at each step.

### Ethical standards

The authors assert that all procedures contributing to this work comply with the ethical standards of the relevant national and institutional committees on human experimentation, and with the Helsinki Declaration of 1975 as revised in 2013. Written informed consent to participate was obtained from all participants. The Omtanke2020 study was approved on 3 June 2020 by the Swedish Ethical Review Authority (no. DNR 2020-01785).

G.L.M. affirms that the manuscript is an honest, accurate and transparent account of the study being reported; that no important aspects of the study have been omitted; and that any discrepancies from the study as planned have been explained.

## Results

### Sample characteristics

**Fig. 1:**
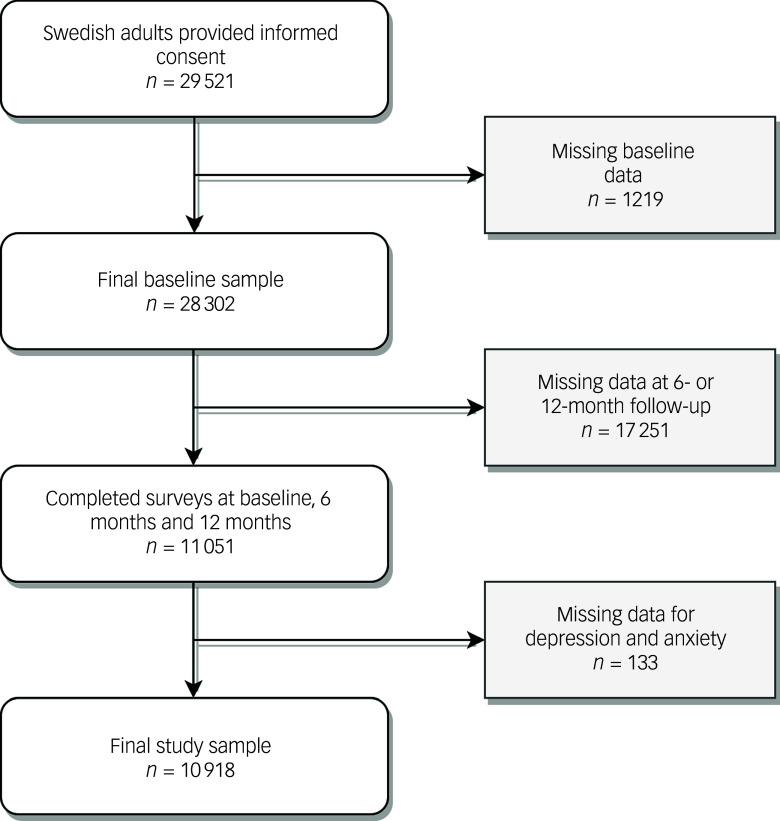
Flowchart of the study sample.

Of the 28 302 participants in the Omtanke2020 study, 11 051 responded to the 6- and 12-month follow-up surveys. Of these participants, 133 had more than 20% missing values for depressive and anxiety symptoms (i.e. above the 20% threshold for imputation). Thus, the final study sample included 10 918 participants (Fig. 1), the majority (82.0%) of whom were female. The mean age at cohort recruitment was similar among men (52.6 (±16.0 s.d.) years) and women (53.3 (±15.3 s.d.) years) (Table 1). Overall, women had healthier self-reported lifestyle factors, lower BMI (54.8% of women in the healthy weight range versus 50.4% of men), lower prevalence of smoking (10.9% of women currently smoking versus 18.1% of men) and fewer chronic diseases (31.1% of women had at least one disease versus 36.4% of men). Conversely, more women reported a history of any mental illness (31.5% of women versus 19.6% of men). Over half (55.4%) of the study sample was recruited through invitation due to participation in other ongoing cohort studies at Karolinska Institute. Characteristics of the original baseline population compared with the current study population are presented in Supplementary Table 2.

**Table 1:** Baseline characteristics of the study sample

Characteristics	Female	Male	Total
*N*	9048 (82.9)	1870 (17.1)	10 918 (100.0)
Age, mean years (s.d.)	53.3 (15.3)	52.6 (16.0)	53.2 (15.4)
Age group (years)			
18–29	781 (8.6)	150 (8.0)	931 (8.5)
30–39	1149 (12.7)	318 (17.0)	1467 (13.4)
40–49	1476 (16.3)	364 (19.5)	1840 (16.9)
50–59	2179 (24.1)	337 (18.0)	2516 (23.0)
60–69	1969 (21.8)	346 (18.5)	2315 (21.2)
≥70	1494 (16.5)	355 (19.0)	1849 (16.9)
Body mass index (kg/m^2^)			
Normal or underweight (<25)	4958 (54.8)	943 (50.4)	5901 (54.0)
Overweight (25–30)	2597 (28.7)	697 (37.3)	3294 (30.2)
Obese (>30)	1089 (12.0)	206 (11.0)	1295 (11.9)
Missing	404 (4.5)	24 (1.3)	428 (3.9)
Physical activity during the past 2 weeks			
Every day	1595 (17.6)	269 (14.4)	1864 (17.1)
4–6 days	3257 (36.0)	604 (32.3)	3861 (35.4)
1–3 days	3065 (33.9)	703 (37.6)	3768 (34.5)
None	1068 (11.8)	286 (15.3)	1354 (12.4)
Missing	63 (0.7)	8 (0.4)	71 (0.7)
CAGE score^a^			
0	6630 (73.3)	1386 (74.1)	8016 (73.4)
1	690 (7.6)	156 (8.3)	846 (7.7)
≥2	470 (5.2)	86 (4.6)	556 (5.1)
Missing	1258 (13.9)	242 (12.9)	1500 (13.7)
Current smoking status			
No	8026 (88.7)	1524 (81.5)	9550 (87.5)
Yes	982 (10.9)	339 (18.1)	1321 (12.1)
Missing	40 (0.4)	7 (0.4)	47 (0.4)
History of mental illness			
No	6090 (67.3)	1493 (79.8)	7583 (69.5)
Yes	2846 (31.5)	366 (19.6)	3212 (29.4)
Missing	112 (1.2)	11 (0.6)	123 (1.1)
Number of physical comorbidities^b^			
None	5923 (65.5)	1127 (60.3)	7050 (64.6)
One	2120 (23.4)	475 (25.4)	2595 (23.8)
Two	530 (5.9)	153 (8.2)	683 (6.3)
Three or more	163 (1.8)	53 (2.8)	216 (2.0)
Missing	312 (3.4)	62 (3.3)	374 (3.4)
Recruitment date			
Jun–Aug 2020	2851 (31.5)	190 (10.2)	3041 (27.9)
Sep–Nov 2020	3543 (39.2)	662 (35.4)	4205 (38.5)
Dec 2020–Feb 2021	2058 (22.7)	786 (42.0)	2844 (26.0)
Mar 2021–Jun 2021	596 (6.6)	232 (12.4)	828 (7.6)
Six-month follow-up date			
Feb–Apr 2021	3991 (44.1)	333 (17.8)	4324 (39.6)
May–Jul 2021	4076 (45.0)	1205 (64.4)	5281 (48.4)
Aug–Nov 2021	981 (10.8)	332 (17.8)	1313 (12.0)
One-year follow-up date			
Dec 2021	7250 (80.1)	1517 (81.1)	8767 (80.3)
Jan 2022	1492 (16.5)	303 (16.2)	1795 (16.4)
Feb 2022	306 (3.4)	50 (2.7)	356 (3.3)
Recruitment type			
Invited	4986 (55.1)	1068 (57.1)	6054 (55.4)
Volunteered	2444 (27.0)	593 (31.7)	3037 (27.8)
Unknown	1618 (17.9)	209 (11.2)	1827 (16.7)

Data are presented as *n* (%), unless otherwise specified.

a. A higher CAGE Substance Abuse Screening Tool score indicates alcohol abuse problems; a total score of ≥2 is considered clinically significant.^19^

b. Includes hypertension, heart disease, respiratory illness (including asthma), chronic kidney failure, cancer, diabetes and impaired immune system, for reasons other than the above.

The prevalence of depressive and anxiety symptoms, social network variables and different covariates across the three time points are summarised in Supplementary Table 3. The prevalence of moderate-to-severe depressive symptoms decreased consistently from baseline to the 12-month follow-up (15.0 to 12.7%), as did the prevalence of anxiety symptoms (9.9 to 8.9%). The proportion of marriage and parenthood remained stable over time (∼72%). While contact frequency varied across the three time points, family-related variables (emotional support, closeness and love) showed a stable pattern. Societal cohesion and helpfulness towards others varied, with an increase in negative feelings toward cohesion over time (baseline, 18.5% strongly disagreeing versus 12-month follow-up, 24.7%). Employment and economic difficulties remained stable while COVID-19-related worries fluctuated, dropping from 30.9 to 16.1% over time.

### SEMs

#### Cross-sectional models

**Fig. 2:**
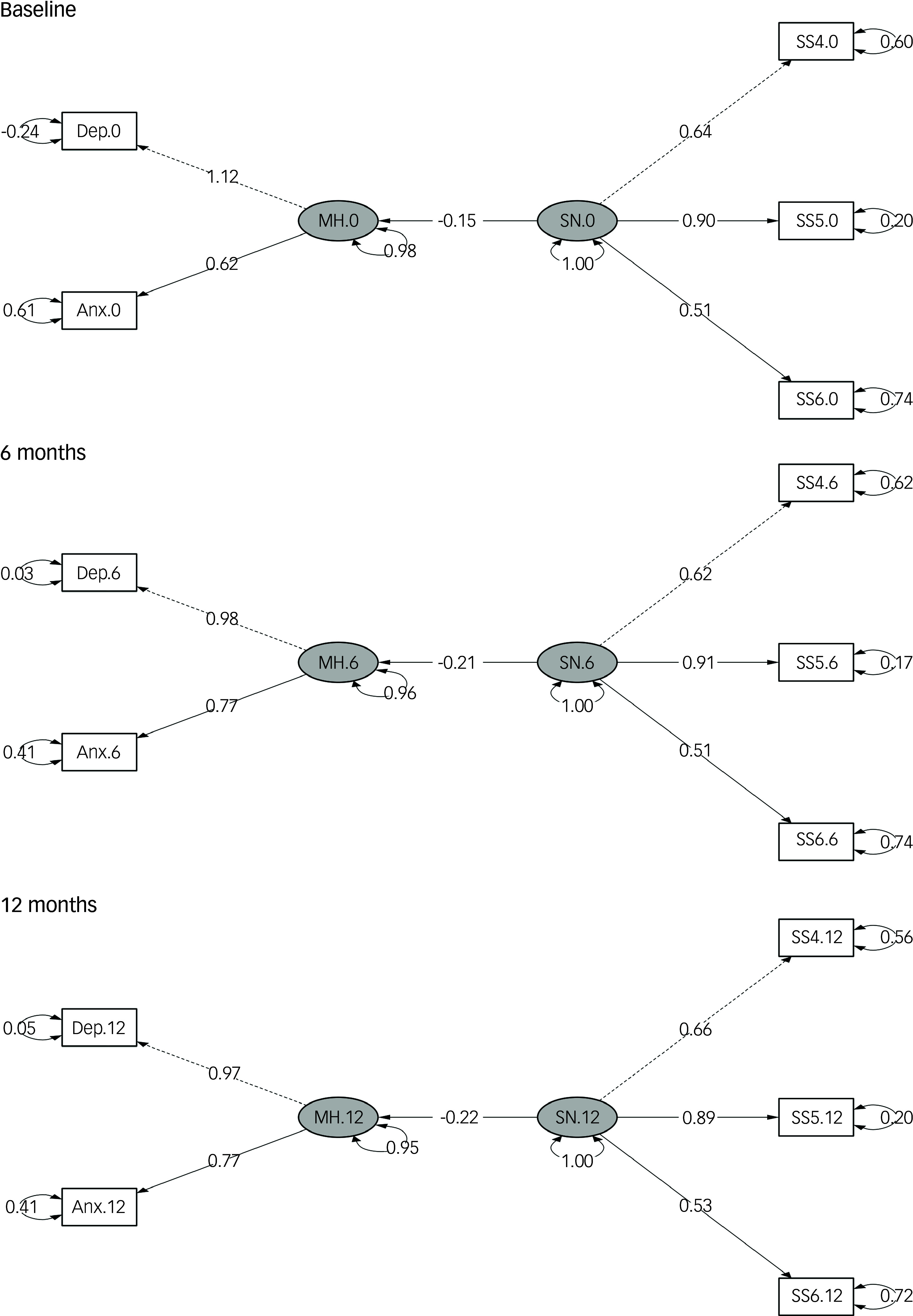
Path diagrams of adjusted cross-sectional models at baseline and 6- and 12-month follow-ups. Anx, anxiety symptoms; Dep, depressive symptoms; MH, mental health; SN, social network; SS4, family closeness; SS5, perceived warmth or love from others; SS6, increased societal cohesion. Dashed lines indicate paths from latent variables to the first observed endogenous variables, which are set to a normalisation constraint of 1.00; solid lines indicate other regression paths between latent variables SN and MH, as well as those between latent and observed variables. Two-headed curved arrows indicate the standardised variance for each observed variable. All paths presented in the diagram are statistically significant. The fully adjusted baseline model was used, adjusting for age, gender, previous psychiatric diagnosis, physical comorbidities, body mass index, smoking status, CAGE Substance Abuse Screening Tool score, physical activity, recruitment type, previous COVID-19 infection, COVID-19-related worries, economic difficulties, employment status and response period. Models for the 6- and 12-month follow-ups were adjusted for previous COVID-19 infection, COVID-19-related worries, economic difficulties, employment status and response period.

Across all time points and models, the three social network indicators consistently demonstrating the best model fit statistics were closeness to family, perceived warmth or love from others and increased societal cohesion; these indicators were therefore used in all final cross-sectional models (see Fig. 2; path diagrams for the unadjusted models are given in Supplementary Fig. 3). The baseline cross-sectional model showed a significant inverse association between social network and mental health (*β* = −0.19, *P* < 0.001), with similar magnitudes at 6 months (*β* = −0.23, *P* < 0.001) and 12 months (*β* = −0.22, *P* < 0.001). Fit statistics for the three cross-sectional models were good (Table 2; *χ* ^2^ significant, while CFI, TLI and RMSEA met conventional thresholds). Coefficients remained stable following stepwise adjustment for covariates (fully adjusted cross-sectional coefficients: model 1, *β* = −0.15; model 2, *β* = −0.14; model 3. *β* = −0.15, all *P* < 0.001), supporting the robustness of the contemporaneous associations.

**Table 2:** Model fit statistics for all structural equation models after trimming procedure applied

Model	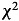	d.f.	CFI	TLI	RMSEA (90% CI)	SRMR
Cross-sectional models						
Unadjusted						
Baseline	99.94***	10	0.995^a^	0.987^a^	0.047 (0.039–0.055)^a^	0.025^a^
Six-month follow-up	116.65***	4	0.994^a^	0.985^a^	0.051 (0.043–0.059)	0.026^a^
One-year follow-up	105.48***	4	0.995^a^	0.987^a^	0.048 (0.041–0.056)^a^	0.024^a^
Adjusted						
Baseline model 1	71.21***	4	0.996^a^	0.990^a^	0.039 (0.032–0.047)^a^	0.020^a^
Baseline model 2	72.42***	4	0.995^a^	0.989^a^	0.040 (0.032–0.048)^a^	0.021^a^
Baseline model 3	47.99***	4	0.997^a^	0.992^a^	0.032 (0.024–0.040)^a^	0.017^a^
Six-month follow-up	52.95***	4	0.997^a^	0.993^a^	0.033 (0.026–0.042)^a^	0.018^a^
One-year follow-up	52.70***	4	0.997^a^	0.993^a^	0.033 (0.026–0.042)^a^	0.015^a^
Cross-lagged models						
Unadjusted	3172.72***	29	0.937	0.903	0.100 (0.097–0.103)	0.039^a^
Adjusted						
Model 1	2933.95***	31	0.932	0.901	0.093 (0.090–0.095)	0.040^a^
Model 2^b^	2841.46***	31	0.928	0.896	0.091 (0.088–0.094)	0.039^a^
Model 3^b^	2713.71***	31	0.931	0.899	0.089 (0.086–0.092)	0.038^a^

CFI, comparative fit index; TLI, Tucker–Lewis index; RMSEA, root mean square error of approximation; SRMR, standardised root mean squared residual.

Model 1: adjusted for age and gender.

Model 2: adjusted additionally for history of mental illness, physical comorbidities, body mass index, smoking status, CAGE Substance Abuse Screening Tool score, physical activity and recruitment type.

Model 3: adjusted additionally for previous COVID-19 infection, COVID-19-related worries, economic difficulties, employment status and response period.

a. Meets the requirement for good model fit.

b. Also adjusted for change in employment.

***P < 0.001.

#### Cross-lagged models

**Fig. 3:**
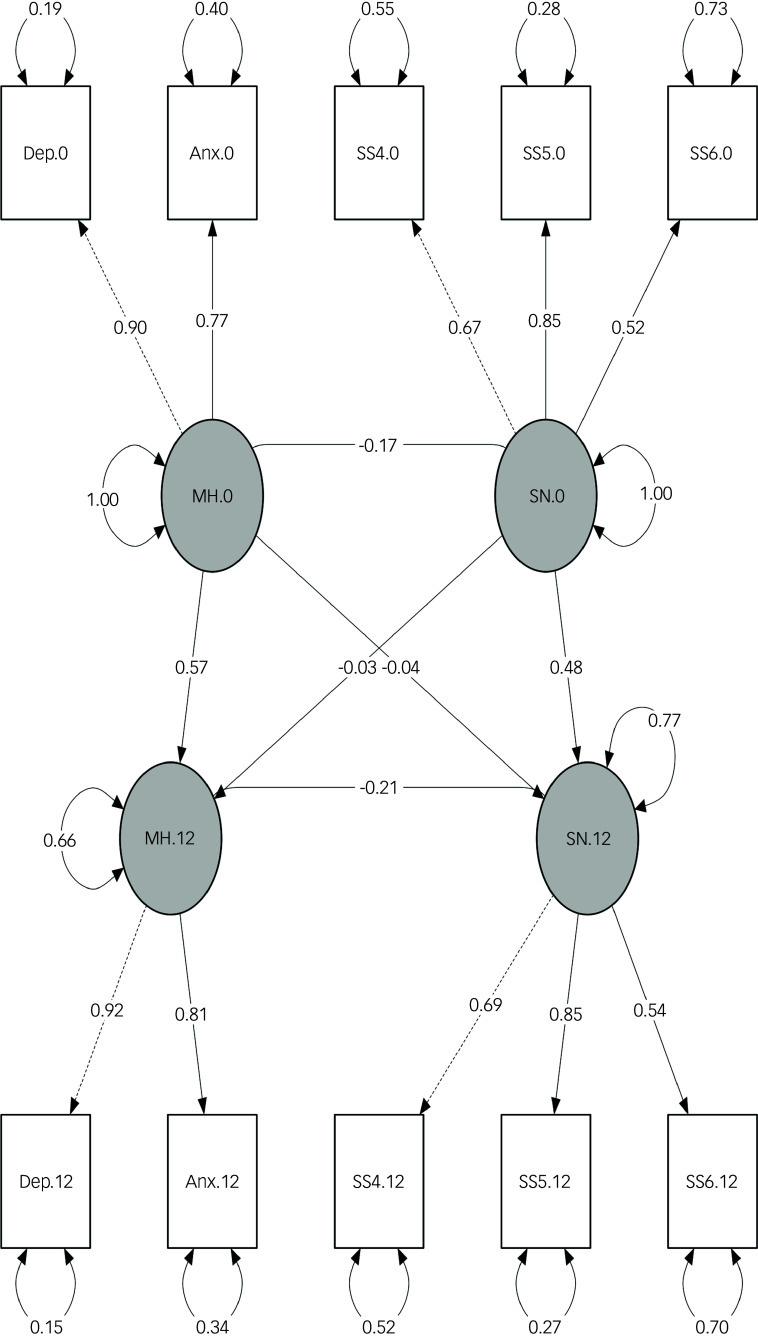
Path diagram of the fully adjusted cross-lagged panel model at baseline (0) and 1-year follow-up (12). Anx, anxiety symptoms; Dep, depressive symptoms; MH, mental health; SN, social network; SS4, family closeness; SS5, perceived warmth or love from others; SS6, increased societal cohesion. Dashed lines indicate paths from latent variables to the first observed endogenous variables, which are set to a normalisation constraint of 1.00; solid lines indicate other regression paths between latent variables SN and MH, as well as those between latent and observed variables. Two-headed curved arrows indicate the standardised variance for each observed variable. All paths presented in the diagram are significant. The fully adjusted model (model 3) was implemented using the following baseline variables: age, gender, previous psychiatric diagnosis, physical comorbidities, body mass index, smoking status, CAGE Substance Abuse Screening Tool score, physical activity, recruitment type, previous COVID-19 infection, COVID-19-related worries, economic difficulties, employment status and response period. The 1-year model is adjusted for previous COVID-19 infection, COVID-19-related worries, economic difficulties, employment status and response period.

Consistent with the cross-sectional models, the latent social network construct defined by family closeness, perceived warmth or love from others and increased societal cohesion demonstrated the best fit in the cross-lagged models. The path diagram for the adjusted cross-lagged analysis is presented in Fig. 3.

In the adjusted model, temporal stability (autoregressive effects) was moderate for both constructs: social network baseline → 1 year, *β* = 0.46, *P* < 0.001; mental health baseline → 1 year, *β* = 0.57, *P* < 0.001. Contemporaneous correlations between social network and mental health were negative at both time points, with slightly weaker baseline association in the adjusted model (*β* = −0.17, *P* < 0.001) than in the unadjusted model (*β* = −0.21, *P* < 0.001) and a slightly stronger correlation at 1 year (*β* = −0.21, *P* < 0.001, adjusted; *β* = −0.15, *P* < 0.001, unadjusted).

During model development, associations at the 6-month wave were consistently weaker; therefore, 6-month measurements were excluded to improve parsimony and model fit. Across specifications, goodness-of-fit statistics for the cross-lagged models were slightly lower than for the cross-sectional models, but overall fit was acceptable for the adjusted 0- to 12-month model (CFI 0.960, TLI 0.931, RMSEA 0.074, SRMR 0.022; see Table 2 for fit indices).

#### Subgroup analysis

Results from the analysis stratified by gender are displayed in Supplementary Tables 4 and 5, while results from the age-stratified analysis are shown in Supplementary Tables 6 and 7. Regardless of gender and age, the same social network variables (family closeness, perceived warmth or love from others and increased societal cohesion) consistently had the strongest correlations with contemporary depressive and anxiety symptoms and best model fit statistics. Gender did not appear to have a consistent impact on mental health or social network (Supplementary Tables 4 and 5), but there was a stronger positive correlation among men than among women between mental health at baseline and that at 1 year (Supplementary Table 5; 0.66, *P* < 0.001 for men versus 0.56, *P* < 0.001 for women). In the age-stratified analysis, participants aged 60 years or older displayed the most significant change in the correlation between social network and mental health over time (Supplementary Table 6; −0.10, *P* < 0.001 at baseline versus −0.20, *P* < 0.001 at 1-year follow-up). All other estimates were relatively stable across age groups. Similarly, the cross-lagged models exhibited worse fit statistics than the cross-sectional ones (Table 2).

## Discussion

To the best of our knowledge, this is the first study to use SEM, including CLPM, to analyse social network and mental health during the COVID-19 pandemic in a large cohort. The cross-sectional and cross-lagged models confirmed the study hypotheses, namely that stronger perceived social support is inversely associated with poor mental health outcomes, with minor gender differences detected. While the prevalence of depressive and anxiety symptoms decreased over the study period, social network variables remained stable predictors of depressive and anxiety symptoms. Furthermore, although the relationship between social network and mental symptoms was stronger at baseline than at the 1-year follow-up, the continued statistical significance of this relationship underscores the enduring influence of perceived social support on mental well-being. Results did not vary greatly by gender or age.

### Results in context

At baseline, the prevalence of moderate-to-severe symptoms was 15.0% for depression (PHQ-9 ≥10) and 9.9% for anxiety (GAD-7 ≥10) (Supplementary Table 3). These rates are broadly consistent with pandemic-era population estimates for Sweden, and exceed many pre-pandemic estimates. For instance, an analysis of data from the European Health Interview Survey (EHIS-3) reported an overall prevalence of clinically relevant depressive symptoms of 6.54% pre-pandemic (2018–2020), with Sweden at approximately 10.72%.^22^ Compared with the full Omtanke2020 cohort, the current analytic sample was older, reported fewer depressive and anxiety symptoms and generally had more favourable socioeconomic and health characteristics (Supplementary Table 2).

Our statistical models started with the inclusion of all 14 social network variables collected in the study, and these variables were systematically removed from the models based on their impact on model fit.^21^ The variables that consistently fit the models and had the strongest correlation with depressive and anxiety symptoms were closeness to family, perceived warmth or love from others and increased societal cohesion. These results align with previous studies showing that emotional, perceived aspects of social network are stronger predictors of mental and physical well-being as opposed to the more quantifiable aspects (e.g. number of interpersonal relationships).^23,24^ Notably, these three social network variables represent different levels of social relationships. Family closeness and perceived warmth or love from others refer to relationships with another individual, while the perception of societal cohesion goes beyond individual relationships and explores the broader fabric of a community or society. Kim et al and Smith et al have reported strong associations between neighbourhood social cohesion and lower rates of negative mental health symptoms.^25,26^

The social network variables that consistently failed to fit the models and had the weakest correlations with mental health outcomes were the presence of other family members in the household and the frequency of contacts with social network members. Household size has often been shown to be strongly associated with mental health,^27,28^ at odds with the results of this study. Objectively measured family structures may not capture the nuanced emotional support and interpersonal dynamics that are crucial for mental well-being.^29^ It is possible, therefore, that the quality of relationships within the household, rather than the objective number of people, plays a more significant role in influencing mental health in the context of a stressful situation such as the COVID-19 pandemic. Although rare, several other studies have also used SEM to explore the relationships between different measures and mental health during the COVID-19 pandemic. Specifically, a small cross-sectional study conducted in Singapore^11^ revealed that compassion was associated with a greater level of pandemic-related protective health behaviours (i.e. hand hygiene and physical distancing), which in turn was negatively associated with anxiety symptoms.

One of the notable characteristics of the COVID-19 pandemic lies in the differential global responses to the pandemic’s challenges. Some governments implemented strict lockdown laws that effectively curbed viral spread in some cases but amplified feelings of social isolation.^6^ Sweden, on the other hand, aimed to balance virus containment with preserving social connectedness, by avoiding stay-at-home orders in favour of voluntary guidelines to stay home and avoid public places in the first wave of the pandemic.^14^ This approach was in direct contrast with other European countries early in the pandemic. For example, Denmark ordered a 2-week stay-at-home order in March 2020^30^ while Norway mandated quarantine periods for travellers returning from abroad.^31^ Ebrahimi et al reported that population-level depressive symptoms in Norway were strongly associated with the presence of strict physical distancing policies, and symptoms decreased with the termination of these policies.^32^ Thus, Sweden’s pandemic experience has provided a valuable case study for assessing the implications of relatively lenient restrictions on social networks and mental health outcomes.

### Strengths and limitations

This study has several strengths. It leverages a large sample and a longitudinal design with three measurement waves, and uses validated instruments to assess depressive and anxiety symptoms. Analytically, we adjusted for a comprehensive set of potential confounders including time-varying covariates and used SEM, which helps account for measurement error and enables estimation of complex direct and indirect relationships and the proportion of variance explained by predictors.^33–35^ Nevertheless, the findings should be interpreted while also considering several limitations. Selection bias is a potential issue, because the overall Omtanke2020 cohort and the analytic sample used in this study consist of a disproportionately higher proportion of older women, which limits the representativeness of our findings to the general Swedish population.^36^ Methodologically, the multiplicity inherent in complex structural equation models could inflate certain associations, and residual confounding (for example, by income or education) cannot be ruled out. Finally, we note as a limitation that the social network items were not derived from a single established validated instrument. Although the items map onto commonly used structural and perceived support domains and show expected factor loadings in SEM, future work using validated multi-item scales or formal psychometric validation would improve measurement validity and thereby strengthen the credibility of the observed associations.

### Public health implications

The COVID-19 pandemic has underscored how reduced social contact can significantly impact mental health, particularly for individuals who are socially isolated yet not engaged with healthcare systems. While our findings confirm the protective role of perceived social support, they also highlight a challenge, namely how to identify and support at-risk individuals who may not meet clinical thresholds or seek treatment.

Future public health strategies should incorporate brief social support screening tools into non-clinical settings – such as primary care, schools, workplaces and digital platforms – to proactively identify those at risk. Interventions such as teletherapy, peer support groups and community-based mutual aid programmes could subsequently be mobilised to provide connection during times of crisis.^37–41^ Sustained investment in community cohesion is especially important for prevention of mental health deterioration during future pandemics or similar disruptions.^42^

While findings in this area may seem difficult to act upon clinically, they hold clear relevance for population-level prevention. Social support remains a modifiable factor, and addressing it requires early, community-centred and cross-sector approaches.

In conclusion, this large cohort study presents a distinctive approach by examining the relationships between social network and depressive and anxiety symptoms across various time points during the COVID-19 pandemic in Sweden. The utilisation of SEM to explore these associations empirically corroborates the protective role of perceived social support in mental health outcomes, and emphasises the importance of fostering strong social networks, especially in times of crisis.

## Supporting information

Murphy et al. supplementary material Murphy et al. supplementary material

## Data Availability

The raw data-sets are unavailable for sharing due to Swedish data protection laws. Statistical code will be available on request to the corresponding author.

## Supplementary material

The supplementary material is available online at https://doi.org/10.1192/bjo.2025.10915

## References

[1] Santini ZI , Koyanagi A , Tyrovolas S , Mason C , Haro JM. The association between social relationships and depression: a systematic review. J Affect Disord 2015; 175: 53–65. 25594512 10.1016/j.jad.2014.12.049

[2] Kawachi I , Berkman LF. Social ties and mental health. J Urban Health 2001; 78: 458–67. 11564849 10.1093/jurban/78.3.458 PMC3455910

[3] Feeney BC , Collins NL. A new look at social support: a theoretical perspective on thriving through relationships. Pers Soc Psychol Rev 2015; 19: 113–47. 25125368 10.1177/1088868314544222 PMC5480897

[4] Morelli SA , Lee IA , Arnn ME , Zaki J. Emotional and instrumental support provision interact to predict well-being. Emotion 2015; 15: 484–93. 26098734 10.1037/emo0000084 PMC4516598

[5] Wang J , Mann F , Lloyd-Evans B , Ma R , Johnson S. Associations between loneliness and perceived social support and outcomes of mental health problems: a systematic review. BMC Psychiatry 2018; 18: 156. 29843662 10.1186/s12888-018-1736-5 PMC5975705

[6] Haug N , Geyrhofer L , Londei A , Dervic E , Desvars-Larrive A , Loreto V , et al. Ranking the effectiveness of worldwide COVID-19 government interventions. Nat Hum Behav 2020; 4: 1303–12. 33199859 10.1038/s41562-020-01009-0

[7] Pfefferbaum B , North CS. Mental health and the Covid-19 pandemic. N Engl J Med 2020; 383: 510–2. 32283003 10.1056/NEJMp2008017

[8] Perez-Brumer A , Balasa R , Doshi A , Brogdon J , Doan T , Oldenburg CE. COVID-19 related shifts in social interaction, connection, and cohesion impact psychosocial health: longitudinal qualitative findings from COVID-19 treatment trial engaged participants. Int J Environ Res Public Health 2022; 19: 10264. 36011898 10.3390/ijerph191610264 PMC9407900

[9] Wright RN , Faul L , Graner JL , Stewart GW , LaBar KS. Psychosocial determinants of anxiety about the COVID-19 pandemic. J Health Psychol 2022; 27: 2344–60. 34348495 10.1177/13591053211030981

[10] Shi L-S-B , Xu RH , Xia Y , Chen D-X , Wang D. The impact of COVID-19-related work stress on the mental health of primary healthcare workers: the mediating effects of social support and resilience. Front Psychol 2022; 12: 800183. 35126252 10.3389/fpsyg.2021.800183 PMC8814425

[11] Lim XY , Yap AC , Mahendran R , Yu J. The interplay between anxiety, fear, protective behaviors, compassion, and resilience among older adults during a COVID-19 lockdown: a structural equation modeling study. Transl Behav Med 2021; 11: 1172–8. 33793946 10.1093/tbm/ibaa143 PMC8139136

[12] Zhu W , Wei Y , Meng X , Li J. The mediation effects of coping style on the relationship between social support and anxiety in Chinese medical staff during COVID-19. BMC Health Serv Res 2020; 20: 1007. 33148229 10.1186/s12913-020-05871-6 PMC7609823

[13] Ludvigsson JF. The first eight months of Sweden’s COVID-19 strategy and the key actions and actors that were involved. Acta Paediatr 2020; 109: 2459–71. 32951258 10.1111/apa.15582 PMC7537539

[14] Ludvigsson JF. How Sweden approached the COVID -19 pandemic: summary and commentary on the National Commission Inquiry. Acta Paediatr 2023; 112: 19–33. 36065136 10.1111/apa.16535 PMC9538368

[15] Lovik Aó , González-Hijón J , Kähler AK , Valdimarsdóttir UA , Frans EM , Magnusson PKE , et al. Mental health indicators in Sweden over a 12-month period during the COVID-19 pandemic – baseline data of the Omtanke2020 Study. J Affect Disord 2023; 322: 108–17. 36379324 10.1016/j.jad.2022.11.004 PMC9657895

[16] Unnarsdóttir AB , Lovik A , Fawns-Ritchie C , Ask H , Kõiv K , Hagen K , et al. Cohort profile: COVIDMENT: COVID-19 cohorts on mental health across six nations. Int J Epidemiol 2022; 51: e108–22. 35020900 10.1093/ije/dyab234 PMC8690101

[17] Kroenke K , Spitzer RL , Williams JBW. The PHQ-9. J Gen Intern Med 2001; 16: 606–13. 11556941 10.1046/j.1525-1497.2001.016009606.x PMC1495268

[18] Spitzer RL , Kroenke K , Williams JBW , Löwe B. A brief measure for assessing generalized anxiety disorder: the GAD-7. Arch Intern Med 2006; 166: 1092–7. 16717171 10.1001/archinte.166.10.1092

[19] Ewing JA. Detecting alcoholism: the CAGE questionnaire. JAMA 1984; 252: 1905–7. 6471323 10.1001/jama.252.14.1905

[20] Hu L , Bentler PM. Cutoff criteria for fit indexes in covariance structure analysis: conventional criteria versus new alternatives. Struct Equ Modeling 1999; 6: 1–55.

[21] Meyers LS , Gamst G , Guarino AJ. Applied Multivariate Research: Design and Interpretation 3rd ed. SAGE, 2017.

[22] Arias-de la Torre J , Vilagut G , Ronaldson A , Bakolis I , Dregan A , Martín V , et al. Prevalence and variability of depressive symptoms in Europe: update using representative data from the second and third waves of the European Health Interview Survey (EHIS-2 and EHIS-3). Lancet Public Health 2023; 8: e889–98. 37898521 10.1016/S2468-2667(23)00220-7

[23] Murphy GL , Beridze G , Vetrano DL , Calderón-Larrañaga A. Social network and severe lower respiratory tract infections in older adults: findings from a Swedish longitudinal population-based study. Int J Infect Dis 2023; 128: 176–83. 36587838 10.1016/j.ijid.2022.12.031

[24] Sun J , Harris K , Vazire S. Is well-being associated with the quantity and quality of social interactions? J Pers Soc Psychol 2020; 119: 1478–96. 31647273 10.1037/pspp0000272

[25] Kim ES , Chen Y , Kawachi I , VanderWeele TJ. Perceived neighborhood social cohesion and subsequent health and well-being in older adults: an outcome-wide longitudinal approach. Health Place 2020; 66: 102420. 32905980 10.1016/j.healthplace.2020.102420 PMC7686282

[26] Smith RJ , Baik S , Lehning AJ , Mattocks N , Cheon JH , Kim K , et al. Residential segregation, social cohesion, and aging in place: health and mental health inequities. Gerontologist 2022; 62: 1289–98. 35666206 10.1093/geront/gnac076

[27] Cheng Y , Zhang L , Wang F , Zhang P , Ye B , Liang Y. The effects of family structure and function on mental health during China’s transition: a cross-sectional analysis. BMC Fam Pract 2017; 18: 59. 28476107 10.1186/s12875-017-0630-4 PMC5420133

[28] Sempungu JK , Choi M , Lee EH , Lee YH. Changes in household size in the Republic of Korea and depression: a temporal analysis. Asia Pac J Public Health 2023; 35: 214–6. 36872615 10.1177/10105395231160340

[29] Umberson D , Montez JK. Social relationships and health: a flashpoint for health policy. J Health Soc Behav 2010; 51: S54–66. 20943583 10.1177/0022146510383501 PMC3150158

[30] Böhm R , Lilleholt L , Meineche JT , Strandsbjerg CF , Windfeld A , Windfeld FC , et al. The COVID-19 snapshot monitoring in Denmark. Samfundsøkonomen 2020; 4: 62–9.

[31] Ministry of Health and Care Services. Timeline: News from Norwegian Ministries about the Coronavirus Disease Covid-19. Ministry of Health and Care Services, 2020 (https://www.regjeringen.no/en/topics/koronavirus-covid-19/timeline-for-news-from-norwegian-ministries-about-the-coronavirus-disease-covid-19/id2692402/ [cited 31 Aug 2023]).

[32] Ebrahimi OV , Bauer DJ , Hoffart A , Johnson SU. A critical period for pandemic adaptation: the evolution of depressive symptomatology in a representative sample of adults across a 17-month period during COVID-19. J Psychopathol Clin Sci 2022; 131: 881–94. 36326629 10.1037/abn0000786

[33] Gefen D , Straub D , Boudreau M-C. Structural equation modeling and regression: guidelines for research practice. Commun Assoc Inf Syst 2000; 4.

[34] Grimm KJ , Ram N , Estabrook R. Growth Modeling: Structural Equation and Multilevel Modeling Approaches. Guilford Press, 2017.

[35] Jeon J. The strengths and limitations of the statistical modeling of complex social phenomenon: focusing on SEM, path analysis, or multiple regression models. World Acad Sci Eng Technol 2015; 9: 1634–42.

[36] Statistics Sweden . Average and Median Age in Sweden by Sex (1968-2024). Statistics Sweden, 2025 (https://www.statistikdatabasen.scb.se/pxweb/en/ssd/START__BE__BE0101__BE0101B/BefolkMedianAlder/ [cited 26 Aug 2025]).

[37] Hubley S , Lynch SB , Schneck C , Thomas M , Shore J. Review of key telepsychiatry outcomes. World J Psychiatry 2016; 6: 269–82. 27354970 10.5498/wjp.v6.i2.269 PMC4919267

[38] Bashshur RL , Shannon GW , Bashshur N , Yellowlees PM. The empirical evidence for telemedicine interventions in mental disorders. Telemed J E Health 2016; 22: 87–113. 26624248 10.1089/tmj.2015.0206 PMC4744872

[39] Augusterfer EF , Mollica RF , Lavelle J. A review of telemental health in international and post-disaster settings. Int Rev Psychiatry 2015; 27: 540–6. 26576720 10.3109/09540261.2015.1082985

[40] Fernandes-Jesus M , Mao G , Ntontis E , Cocking C , McTague M , Schwarz A , et al. More than a COVID-19 response: sustaining mutual aid groups during and beyond the pandemic. Front Psychol 2021; 12: 716202. 34744875 10.3389/fpsyg.2021.716202 PMC8563598

[41] El Zerbi C , Hartopp N , Ramsay A , Marlow S. More tangible and less theoretical: understandings and experiences of neighbourhood-led Mutual Aid groups during the COVID-19 pandemic. J Civ Soc 2022; 18: 453–67.

[42] Svensson SJ , Elntib S. Community cohesion during the first peak of the COVID-19 pandemic: a social antidote to health anxiety and stress. J Appl Soc Psychol 2021; 51: 793–808. 34219802 10.1111/jasp.12800 PMC8237056

