# Supplementary material for: The influence of social network on depressive and anxiety symptoms during the COVID-19 pandemic: findings from a Swedish cohort study
Source: BJPsych Open. 2025 Dec 23;12(1):e26. doi: 10.1192/bjo.2025.10915 (PMC12724092; doi:10.1192/bjo.2025.10915)
Supplement: Murphy et al. supplementary material [file S2056472425109150sup001.docx]

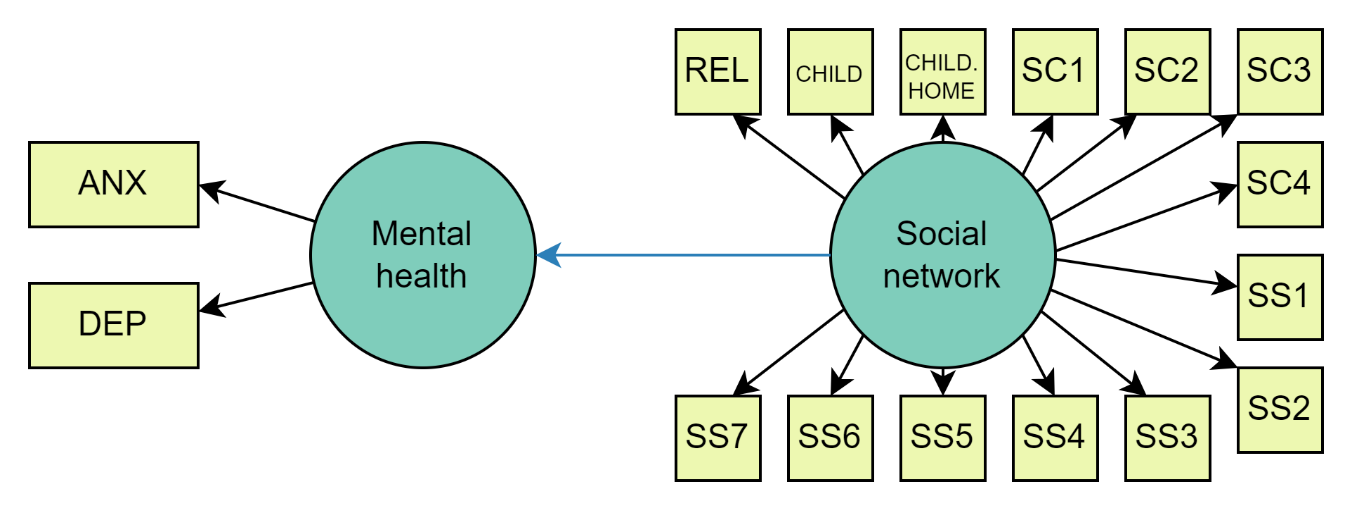


**Supplementary Figure 1.** Hypothesized cross-sectional structural equation model. ANX = anxiety symptoms; DEP = depressive symptoms; REL = relationship status; CHILD = children; CHILD. HOME = children living at home; SC1 = phone contact; SC2 = social media contact; SC3 = in-person contact; SC4 = left home; SS1 = emotional support from family; SS2 = emotional support from a specific person; SS3 = feeling safe at home; SS4 = family closeness; SS5 = perceived warmth or love from others; SS6 = increased societal cohesion; SS7 = feeling helpful during a crisis.


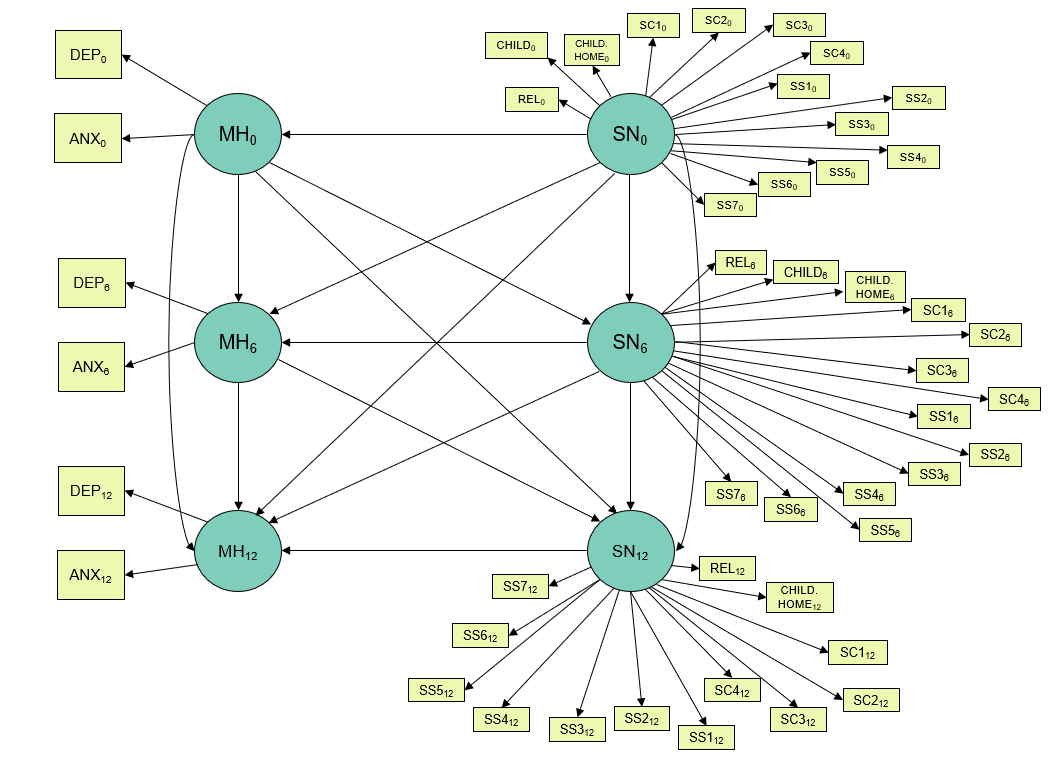


**Supplementary Figure 2.** Hypothesized cross-lagged panel model at baseline (0), six-month follow-up (6), and 12-month follow-up (12). ANX = anxiety symptoms; DEP = depressive symptoms; MH = mental health; SN = social network; REL = relationship status; CHILD = children; CHILD. HOME = children living at home; SC1 = phone contact; SC2 = social media contact; SC3 = in-person contact; SC4 = left home; SS1 = emotional support from family; SS2 = emotional support from a specific person; SS3 = feeling safe at home; SS4 = family closeness; SS5 = perceived warmth or love from others; SS6 = increased societal cohesion; SS7 = feeling helpful during the crisis.


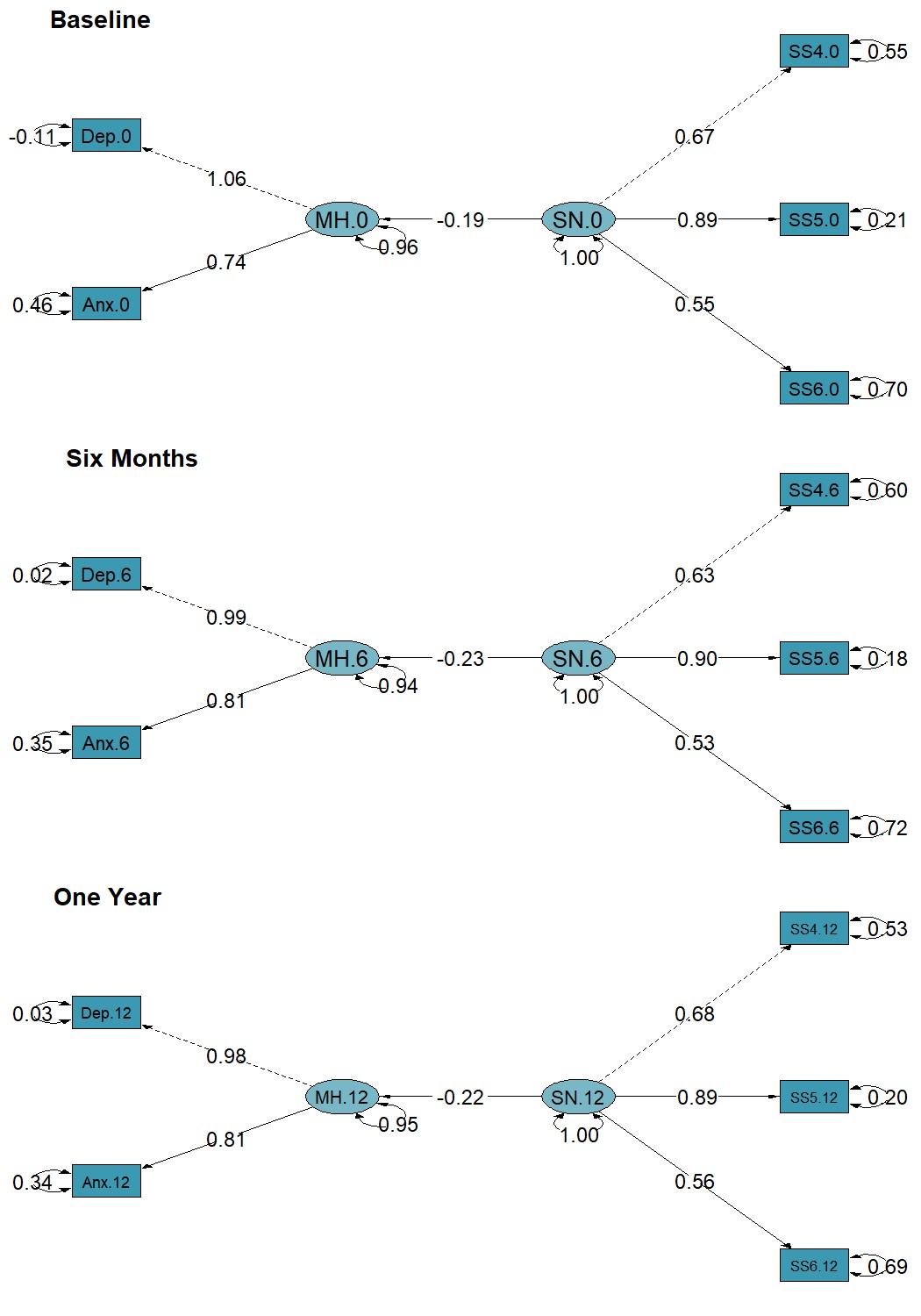


**Supplementary Figure 3. Path diagrams of unadjusted cross-sectional models at baseline (0), six-month follow-up (6), and one-year follow-up (12). ANX = anxiety symptoms; DEP = depressive symptoms; MH = mental health; SN = social network; SS4 = family closeness; SS5 = perceived warmth or love from others; SS6 = increased societal cohesion. Dashed lines indicate paths from latent variables to the first observed endogenous variables, which are set to a normalization constraint of 1.00; solid lines indicate other regression paths between latent variables SN and MH, as well as regression paths between latent variables and observed variables. Two-headed curved arrows indicate the standardized variance for each observed variable. All paths presented in the diagram are statistically significant.**


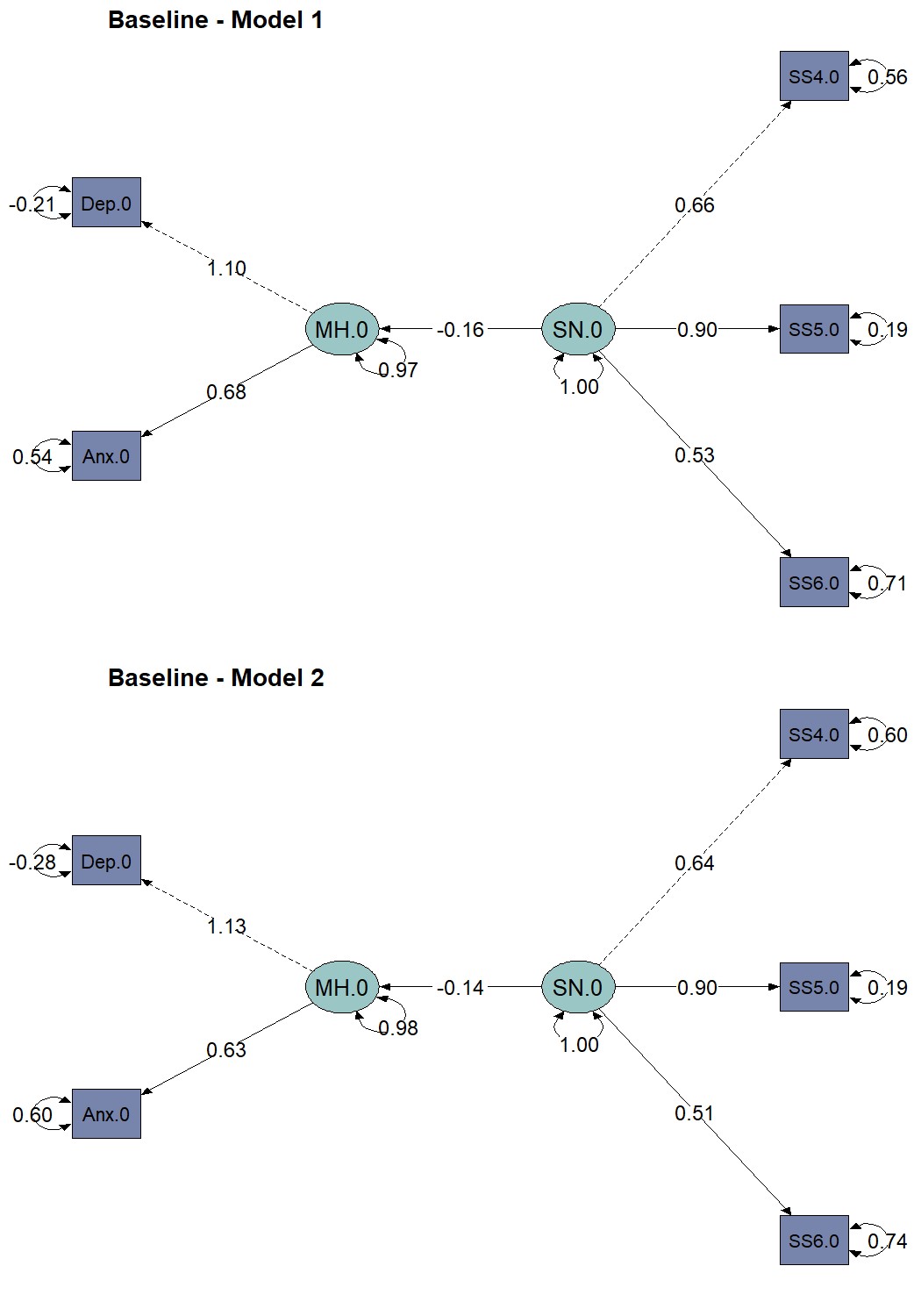


**Supplementary Figure 4. Path diagrams of partially adjusted baseline cross-sectional models. ANX = anxiety symptoms; DEP = depressive symptoms; MH = mental health; SN = social network; SS4 = family closeness; SS5 = perceived warmth or love from others; SS6 = increased societal cohesion. Dashed lines indicate paths from latent variables to the first observed endogenous variables, which are set to a normalization constraint of 1.00; solid lines indicate other regression paths between latent variables SN and MH, as well as regression paths between latent variables and observed variables. Two-headed curved arrows indicate the standardized variance for each observed variable. All paths presented in the diagram are statistically significant. Model 1 has been adjusted for age and sex, while Model 2 has been additionally adjusted for previous psychiatric diagnosis, physical comorbidities, BMI, smoking status, CAGE score, physical activity, and recruitment type.**


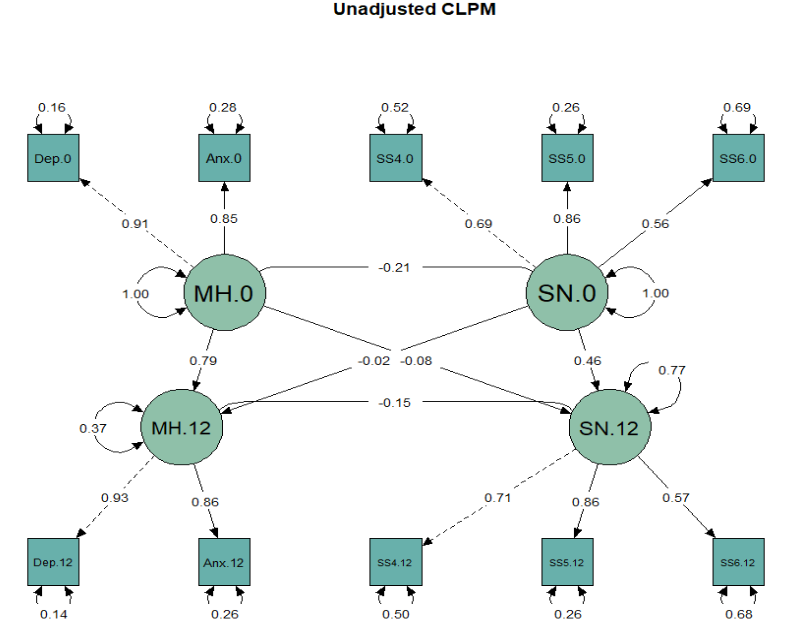


**Supplementary Figure 5.** Path diagram of the unadjusted cross-lagged panel model (CLPM) at baseline (0) and one-year follow-up (12). ANX = anxiety symptoms; DEP = depressive symptoms; MH = mental health; SN = social network; SS4 = family closeness; SS5 = perceived warmth or love from others; SS6 = increased societal cohesion. Dashed lines indicate paths from latent variables to the first observed endogenous variables, which are set to a normalization constraint of 1.00; solid lines indicate other regression paths between latent variables SN and MH, as well as regression paths between latent variables and observed variables. Two-headed curved arrows indicate the standardized variance for each observed variable. All paths presented in the diagram are statistically significant.

**Supplementary Table 1.** Description of the social network items included in the baseline, six-month, and one-year follow-up surveys.

| Category | Variable Name | Questions | Possible Answers |
| --- | --- | --- | --- |
| Family | REL | What is your current relationship status? | Married; cohabitating or other firm relationship; single or divorced; widow |
|  | CHILD | Do you have children?^†^ | Yes; no |
|  | CHILD.HOME | Does/do your child/any of your children in your household live at home? | Yes; no |
| Frequency of social contact |  | In the last two weeks, how often have you… | Several times per day; once per day; 5-6 days per week; 3-4 days per week; 1-2 days per week; less than once per week |
|  | SC1 | 1. Spoken with your family members, relatives, friends, or neighbors on the phone? |  |
|  | SC2 | 2. Been in contact with your family, relatives, friends, or neighbors via social media? |  |
|  | SC3 | 3. Met your family, relatives, or friends in person? |  |
|  | SC4 | 4. Left your home? |  |
| Perceived Social support |  | Do you agree or disagree with the following statements when you think of the last two months? | Do not agree at all; do not agree; do not agree nor disagree; agree a lot; completely agree |
|  | SS1 | 1. I get the emotional support and help I need from my family. |  |
|  | SS2 | 2. I have a specific person I can turn to when I need comforting. |  |
|  | SS3 | 3. I feel safe in my home. |  |
|  | SS4 | 4. My family is closer to me than before. |  |
|  | SS5 | 5. I have felt more warmth/love from others than before. |  |
|  | SS6 | 6. I feel that the cohesion in society has increased. |  |
|  | SS7 | 7. I feel that I have been helpful to others in the current situation. |  |

^†^Participants were not asked this question at the one-year follow-up.

**Supplementary Table 2.** Characteristics of participants excluded due to missing social network and/or mental health data (N = 17,384) compared with the final analytic sample (N = 10,918).

| **Characteristic** | **Excluded N = 17,384** | **Final sample N = 10,918** | **p-value^1^** |
| --- | --- | --- | --- |
| **Age, mean (SD)** | 46 (15) | 53 (15) | <0.001 |
| **Sex** |  |  | <0.001 |
| Female | 14,023 (81%) | 9,048 (83%) |  |
| Male | 3,361 (19%) | 1,870 (17%) |  |
| **Age group** |  |  | <0.001 |
| 18-29 | 2,999 (17%) | 931 (8.5%) |  |
| 30-39 | 3,751 (22%) | 1,467 (13%) |  |
| 40-49 | 3,570 (21%) | 1,840 (17%) |  |
| 50-59 | 3,529 (20%) | 2,516 (23%) |  |
| 60-69 | 2,147 (12%) | 2,315 (21%) |  |
| ≥70 | 1,388 (8.0%) | 1,849 (17%) |  |
| **Depressive symptoms** |  |  |  |
| Mean (SD) | 6.5 (5.8) | 4.8 (4.8) | <0.001 |
| Above cutoff^2^ | 4,290 (25%) | 1,634 (15%) | <0.001 |
| Missing | 405 | 0 |  |
| **Anxiety symptoms** |  |  |  |
| Mean (SD) | 5.0 (5.0) | 3.5 (4.2) | <0.001 |
| Above cutoff^3^ | 3,083 (18%) | 1,085 (9.9%) | <0.001 |
| Missing | 405 | 0 |  |
| **Relationship** |  |  | 0.2 |
| Single/divorced or widowed | 4,691 (27%) | 2,968 (27%) |  |
| Married or cohabitating | 12,594 (72%) | 7,905 (72%) |  |
| Missing | 99 (0.6%) | 45 (0.4%) |  |
| **Children** |  |  | <0.001 |
| No | 5,668 (33%) | 3,001 (27%) |  |
| Yes | 11,690 (67%) | 7,911 (72%) |  |
| Missing | 26 (0.1%) | 6 (<0.1%) |  |
| **Children at home** |  |  | <0.001 |
| No | 10,244 (59%) | 7,535 (69%) |  |
| Yes | 7,108 (41%) | 3,368 (31%) |  |
| Missing | 32 (0.2%) | 15 (0.1%) |  |
| **Employment** |  |  | <0.001 |
| Full-time work | 7,895 (45%) | 5,197 (48%) |  |
| Part-time work | 1,036 (6.0%) | 634 (5.8%) |  |
| Not working | 1,325 (7.6%) | 696 (6.4%) |  |
| Retired | 2,126 (12%) | 3,036 (28%) |  |
| Student | 1,269 (7.3%) | 489 (4.5%) |  |
| Missing | 3,733 (21%) | 866 (7.9%) |  |
| **Economic difficulties** |  |  | <0.001 |
| Very easy | 10,161 (58%) | 7,693 (70%) |  |
| Quite easy | 3,310 (19%) | 1,704 (16%) |  |
| Neither/nor | 2,443 (14%) | 1,149 (11%) |  |
| Quite difficult | 1,001 (5.8%) | 274 (2.5%) |  |
| Very difficult | 353 (2.0%) | 81 (0.7%) |  |
| Missing | 116 (0.7%) | 17 (0.2%) |  |
| **Previous COVID-19 infection** |  |  | <0.001 |
| No | 5,494 (32%) | 2,459 (23%) |  |
| Yes | 1,064 (6.1%) | 377 (3.5%) |  |
| Missing | 10,749 (62%) | 8,046 (74%) |  |
| **COVID-19-related worry** |  |  | <0.001 |
| Very much | 1,720 (9.9%) | 757 (6.9%) |  |
| Somewhat | 4,167 (24%) | 2,618 (24%) |  |
| Neither/nor | 2,928 (17%) | 1,953 (18%) |  |
| A little | 5,550 (32%) | 4,029 (37%) |  |
| Not at all | 2,452 (14%) | 1,540 (14%) |  |
| Missing | 567 (3.3%) | 21 (0.2%) |  |
| **History of mental illness** |  |  | <0.001 |
| No | 10,229 (59%) | 7,583 (69%) |  |
| Yes | 6,262 (36%) | 3,212 (29%) |  |
| Missing | 893 (5.1%) | 123 (1.1%) |  |
| **Number of physical comorbidities** |  |  | <0.001 |
| None | 11,129 (64%) | 7,050 (65%) |  |
| One | 3,649 (21%) | 2,595 (24%) |  |
| Two | 972 (5.6%) | 683 (6.3%) |  |
| Three or more | 340 (2.0%) | 216 (2.0%) |  |
| Missing | 1,294 (7.4%) | 374 (3.4%) |  |
| **Body mass index (kg/m^2^)** |  |  |  |
| Mean (SD) | 25.6 (5.0) | 25.2 (4.4) | <0.001 |
| Under or normal weight (<25) | 8,563 (49%) | 5,901 (54%) |  |
| Overweight (25-30) | 4,899 (28%) | 3,294 (30%) |  |
| Obese (>30) | 2,480 (14%) | 1,295 (12%) |  |
| Missing | 1,442 (8.3%) | 428 (3.9%) |  |
| **Physical activity during the past two weeks** |  |  | <0.001 |
| Every day | 2,448 (14%) | 1,864 (17%) |  |
| 4-6 days | 5,229 (30%) | 3,861 (35%) |  |
| 1-3 days | 6,387 (37%) | 3,768 (35%) |  |
| None | 2,519 (14%) | 1,354 (12%) |  |
| Missing | 801 (4.6%) | 71 (0.7%) |  |
| **Currently smoking** |  |  | <0.001 |
| No | 13,271 (76%) | 9,550 (87%) |  |
| Yes | 3,337 (19%) | 1,321 (12%) |  |
| Missing | 776 (4.5%) | 47 (0.4%) |  |
| **CAGE score** |  |  | <0.001 |
| 0 | 11,656 (67%) | 8,016 (73%) |  |
| 1 | 1,297 (7.5%) | 846 (7.7%) |  |
| ≥2 | 1,044 (6.0%) | 556 (5.1%) |  |
| Missing | 3,387 (19%) | 1,500 (14%) |  |

*^1^* Wilcoxon rank sum test; Pearson’s Chi-squared test

*^2^* A score of ≥10 on the PHQ-9 indicates moderate-to-severe depressive symptoms.

*^3^* A score of ≥10 on the GAD-7 indicates moderate-to-severe anxiety symptoms.

**Supplementary Table 3. Changes in mental health, social network, and time-varying covariates (N=10,918)**

|  | Baseline | Six-month follow-up | One-year follow-up |
| --- | --- | --- | --- |
| **Mental Health** |  |  |  |
| **Depressive symptoms** |  |  |  |
| Below cutoff | 9,284 (85.0%) | 9,450 (86.6%) | 9,535 (87.3%) |
| Above cutoff | 1,634 (15.0%) | 1,468 (13.4%) | 1,383 (12.7%) |
| Mean (SD) | 4.77 (4.85) | 4.43 (4.81) | 4.25 (4.76) |
| Median [min, max] | 3 [0, 27] | 3 [0, 27] | 3 [0, 27] |
| **Anxiety symptoms** |  |  |  |
| Below cutoff | 9,833 (90.1%) | 9,880 (90.5%) | 9,944 (91.1%) |
| Above cutoff | 1,085 (9.9%) | 1,038 (9.5%) | 974 (8.9%) |
| Mean (SD) | 3.49 (4.19) | 3.40 (4.22) | 3.16 (4.13) |
| Median [min, max] | 2 [0, 21] | 2 [0, 21] | 2 [0, 21] |
|  |  |  |  |
| **Social network** |  |  |  |
| **Relationship** |  |  |  |
| Single/divorced or widowed | 2,968 (27.2%) | 2,994 (27.4%) | 2,999 (27.5%) |
| Married or cohabitating | 7,905 (72.4%) | 7,880 (72.2%) | 7,881 (72.2%) |
| Missing | 45 (0.4%) | 44 (0.4%) | 38 (0.3%) |
| **Children** |  |  |  |
| No | 3,001 (27.5%) | 2,972 (27.2%) | - |
| Yes | 7,911 (72.5%) | 7,934 (72.7%) | - |
| Missing | 6 (0.1%) | 12 (0.1%) | - |
| **Children at home** |  |  |  |
| No | 7,535 (69.0%) | 7,564 (69.3%) | 7,701 (70.5%) |
| Yes | 3,368 (30.8%) | 3,337 (30.6%) | 3,202 (29.3%) |
| Missing | 15 (0.1%) | 17 (0.2%) | 15 (0.1%) |
| **Phone contact** |  |  |  |
| Less than once a week | 1,159 (10.6%) | 1,267 (11.6%) | 1,210 (11.1%) |
| 1-2 days a week | 2,513 (23.0%) | 2,619 (24.0%) | 2,530 (23.2%) |
| 3-4 days a week | 1,998 (18.3%) | 1,988 (18.2%) | 1,974 (18.1%) |
| 5-6 days a week | 1,394 (12.8%) | 1,330 (12.2%) | 1,334 (12.2%) |
| Once a day | 1,739 (15.9%) | 1,842 (16.9%) | 1,778 (16.3%) |
| Several times a day | 2,056 (18.8%) | 1,837 (16.8%) | 2,030 (18.6%) |
| Missing | 59 (0.5%) | 35 (0.3%) | 62 (0.6%) |
| **Social media contact** |  |  |  |
| Less than once a week | 1,454 (13.3%) | 1,512 (13.8%) | 1,400 (12.8%) |
| 1-2 days a week | 1,483 (13.6%) | 1,607 (14.7%) | 1,543 (14.1%) |
| 3-4 days a week | 1,616 (14.8%) | 1,660 (15.2%) | 1,673 (15.3%) |
| 5-6 days a week | 1,432 (13.1%) | 1,476 (13.5%) | 1,409 (12.9%) |
| Once a day | 1,439 (13.2%) | 1,390 (12.7%) | 1,445 (13.2%) |
| Several times a day | 3,226 (29.5%) | 3,012 (27.6%) | 3,152 (28.9%) |
| Missing | 268 (2.5%) | 261 (2.4%) | 296 (2.7%) |
| **In-person contact** |  |  |  |
| Less than once a week | 4,533 (41.5%) | 4,602 (42.2%) | 3,487 (31.9%) |
| 1-2 days a week | 2,835 (26.0%) | 2,935 (26.9%) | 3,268 (29.9%) |
| 3-4 days a week | 1,113 (10.2%) | 1,140 (10.4%) | 1,379 (12.6%) |
| 5-6 days a week | 471 (4.3%) | 482 (4.4%) | 592 (5.4%) |
| Once a day | 466 (4.3%) | 454 (4.2%) | 587 (5.4%) |
| Several times a day | 1,370 (12.5%) | 1,180 (10.8%) | 1,492 (13.7%) |
| Missing | 130 (1.2%) | 125 (1.1%) | 113 (1.0%) |
| **Left home** |  |  |  |
| Less than once a week | 209 (1.9%) | 203 (1.9%) | 101 (0.9%) |
| 1-2 days a week | 812 (7.4%) | 799 (7.3%) | 518 (4.7%) |
| 3-4 days a week | 1,119 (10.2%) | 1,059 (9.7%) | 1,066 (9.8%) |
| 5-6 days a week | 1,463 (13.4%) | 1,375 (12.6%) | 1,627 (14.9%) |
| Once a day | 3,232 (29.6%) | 3,414 (31.3%) | 3,420 (31.3%) |
| Several times a day | 4,001 (36.6%) | 4,014 (36.8%) | 4,129 (37.8%) |
| Missing | 82 (0.8%) | 54 (0.5%) | 57 (0.5%) |
| **Emotional support from family** |  |  |  |
| Strongly disagree | 322 (2.9%) | 330 (3.0%) | 353 (3.2%) |
| Disagree | 1,032 (9.5%) | 1,077 (9.9%) | 1,083 (9.9%) |
| Do not agree nor disagree | 1,291 (11.8%) | 1,253 (11.5%) | 1,087 (10.0%) |
| Agree | 3,080 (28.2%) | 3,227 (29.6%) | 3,327 (30.5%) |
| Strongly agree | 5,072 (46.5%) | 4,914 (45.0%) | 4,920 (45.1%) |
| Missing | 121 (1.1%) | 117 (1.1%) | 148 (1.4%) |
| **Emotional support from specific person** |  |  |  |
| Strongly disagree | 742 (6.8%) | 749 (6.9%) | 748 (6.9%) |
| Disagree | 1,096 (10.0%) | 1,086 (9.9%) | 1,036 (9.5%) |
| Do not agree nor disagree | 992 (9.1%) | 1,083 (9.9%) | 915 (8.4%) |
| Agree | 2,464 (22.6%) | 2,645 (24.2%) | 2,772 (25.4%) |
| Strongly agree | 5,502 (50.4%) | 5,226 (47.9%) | 5,303 (48.6%) |
| Missing | 122 (1.1%) | 129 (1.2%) | 144 (1.3%) |
| **Safety at home** |  |  |  |
| Strongly disagree | 47 (0.4%) | 62 (0.6%) | 68 (0.6%) |
| Disagree | 145 (1.3%) | 137 (1.3%) | 143 (1.3%) |
| Do not agree nor disagree | 216 (2.0%) | 256 (2.3%) | 221 (2.0%) |
| Agree | 1,434 (13.1%) | 1,581 (14.5%) | 1,787 (16.4%) |
| Strongly agree | 9,027 (82.7%) | 8,851 (81.1%) | 8,648 (79.2%) |
| Missing | 49 (0.4%) | 31 (0.3%) | 51 (0.5%) |
| **Family closeness** |  |  |  |
| Strongly disagree | 1,158 (10.6%) | 1,010 (9.3%) | 972 (8.9%) |
| Disagree | 789 (7.2%) | 727 (6.7%) | 678 (6.2%) |
| Do not agree nor disagree | 5,169 (47.3%) | 5,300 (48.5%) | 5,420 (49.6%) |
| Agree | 2,035 (18.6%) | 2,175 (19.9%) | 2,122 (19.4%) |
| Strongly agree | 1,654 (15.1%) | 1,595 (14.6%) | 1,608 (14.7%) |
| Missing | 113 (1.0%) | 111 (1.0%) | 118 (1.1%) |
| **Love from others** |  |  |  |
| Strongly disagree | 1,703 (15.6%) | 1,631 (14.9%) | 1,495 (13.7%) |
| Disagree | 1,361 (12.5%) | 1,153 (10.6%) | 1,080 (9.9%) |
| Do not agree nor disagree | 5,291 (48.5%) | 5,663 (51.9%) | 5,883 (53.9%) |
| Agree | 1,709 (15.7%) | 1,659 (15.2%) | 1,616 (14.8%) |
| Strongly agree | 745 (6.8%) | 715 (6.5%) | 712 (6.5%) |
| Missing | 109 (1.0%) | 97 (0.9%) | 132 (1.2%) |
| **Societal cohesion** |  |  |  |
| Strongly disagree | 2,021 (18.5%) | 2,550 (23.4%) | 2,702 (24.7%) |
| Disagree | 2,292 (21.0%) | 1,802 (16.5%) | 1,695 (15.5%) |
| Do not agree nor disagree | 4,330 (39.7%) | 4,899 (44.9%) | 5,067 (46.4%) |
| Agree | 1,681 (15.4%) | 1,115 (10.2%) | 947 (8.7%) |
| Strongly agree | 344 (3.2%) | 270 (2.5%) | 225 (2.1%) |
| Missing | 250 (2.3%) | 282 (2.6%) | 282 (2.6%) |
| **Helpful to others** |  |  |  |
| Strongly disagree | 1,036 (9.5%) | 986 (9.0%) | 965 (8.8%) |
| Disagree | 2,642 (24.2%) | 2,526 (23.1%) | 2,480 (22.7%) |
| Do not agree nor disagree | 3,370 (30.9%) | 3,790 (34.7%) | 4,033 (36.9%) |
| Agree | 2,574 (23.6%) | 2,498 (22.9%) | 2,387 (21.9%) |
| Strongly agree | 1,130 (10.3%) | 948 (8.7%) | 819 (7.5%) |
| Missing | 166 (1.5%) | 170 (1.6%) | 234 (2.1%) |
|  |  |  |  |
| **Time-varying covariates** |  |  |  |
| **Employment** |  |  |  |
| Full-time work | 5,197 (47.6%) | 5,662 (51.9%) | 5,676 (52.0%) |
| Part-time work | 634 (5.8%) | 575 (5.3%) | 578 (5.3%) |
| Not working | 696 (6.4%) | 758 (6.9%) | 646 (5.9%) |
| Retired | 3,036 (27.8%) | 3,229 (29.6%) | 3,348 (30.7%) |
| Student | 489 (4.5%) | 557 (5.1%) | 550 (5.0%) |
| Missing | 866 (7.9%) | 137 (1.3%) | 120 (1.1%) |
| **Economic difficulties** |  |  |  |
| Very easy | 7,693 (70.5%) | 7,428 (68.0%) | 7,041 (64.5%) |
| Quite easy | 1,704 (15.6%) | 1,631 (14.9%) | 1,839 (16.8%) |
| Neither/nor | 1,149 (10.5%) | 1,408 (12.9%) | 1,582 (14.5%) |
| Quite difficult | 274 (2.5%) | 316 (2.9%) | 310 (2.8%) |
| Very difficult | 81 (0.7%) | 95 (0.9%) | 96 (0.9%) |
| Missing | 17 (0.2%) | 40 (0.4%) | 50 (0.5%) |
| **Previous COVID-19 infection*** |  |  |  |
| No | 2,459 (22.5%) | 3,983 (36.5%) | 3,456 (31.7%) |
| Yes | 377 (3.5%) | 803 (7.4%) | 366 (3.4%) |
| Missing | 8,082 (74.0%) | 6,132 (56.2%) | 7,096 (65.0%) |
| **COVID-19 worry** |  |  |  |
| Very much | 757 (6.9%) | 597 (5.5%) | 295 (2.7%) |
| Somewhat | 2,618 (24.0%) | 1,889 (17.3%) | 1,464 (13.4%) |
| Neither/nor | 1,953 (17.9%) | 1,596 (14.6%) | 1,606 (14.7%) |
| A little | 4,029 (36.9%) | 4,257 (39.0%) | 4,772 (43.7%) |
| Not at all | 1,540 (14.1%) | 2,567 (23.5%) | 2,759 (25.3%) |
| Missing | 21 (0.2%) | 12 (0.1%) | 22 (0.2%) |
| **Employment change** |  |  |  |
| No change | - | 5,625 (51.5%) | 5,980 (54.8%) |
| Not relevant | - | 2,077 (19.0%) | 2,193 (20.1%) |
| Lost job or other negative change | - | 933 (8.5%) | 544 (5.0%) |
| Positive change | - | 1,242 (11.4%) | 1,346 (12.3%) |
| Other | - | 984 (9.0%) | 800 (7.3%) |
| Missing | - | 57 (0.5%) | 55 (0.5%) |

***Participants were asked at six-month and one-year follow-ups if they had tested positive for COVID-19 since the last time they took the survey.**

**Supplementary Table 4. Results from sex-specific analysis showing standardized regression coefficients and model evaluation statistics for cross-sectional structural equation models (women: N=9048; men: N=1870).**

|  | **Baseline** | | **Six-month follow-up** | | **One-year follow-up** | |
| --- | --- | --- | --- | --- | --- | --- |
|  | **Women** | **Men** | **Women** | **Men** | **Women** | **Men** |
| Mental health **🡪** Depression | 1.10* | 1.25* | 0.99* | 0.95* | 0.97* | 1.01* |
| Mental health **🡪** Anxiety | 0.63*** | 0.57*** | 0.76*** | 0.82*** | 0.78*** | 0.74*** |
| Social network **🡪** Family closeness | 0.63* | 0.67* | 0.61* | 0.65* | 0.67* | 0.65* |
| Social network **🡪** Warmth from others | 0.90*** | 0.85*** | 0.91*** | 0.91*** | 0.89*** | 0.91*** |
| Social network **🡪** Societal cohesion | 0.51*** | 0.50*** | 0.51*** | 0.50*** | 0.54*** | 0.49*** |
| Mental health **🡪** Social network | -0.15*** | -0.12*** | -0.20*** | -0.25*** | -0.22*** | -0.22*** |
| **Model Fit** |  |  |  |  |  |  |
| $\boldsymbol{\chi}^{\boldsymbol{2}}$/df | 32.45/4*** | 26.45/4*** | 45.55/4*** | 11.81/4 | 54.92/4*** | 9.08/4 |
| CFI | 0.998^†^ | 0.991^†^ | 0.997^†^ | 0.997^†^ | 0.996^†^ | 0.998^†^ |
| TLI | 0.994^†^ | 0.978^†^ | 0.992^†^ | 0.994^†^ | 0.991^†^ | 0.996^†^ |
| RMSEA | 0.028^†^ | 0.055 | 0.034^†^ | 0.032^†^ | 0.038^†^ | 0.026^†^ |
| SRMR | 0.015^†^ | 0.030^†^ | 0.018^†^ | 0.021^†^ | 0.016^†^ | 0.019^†^ |

*** Coefficients on paths from latent variables to first observed endogenous variables set to normalization constraint of 1 and therefore do not have a p value.**

***** p < 0.001**

**Model 3 was used for baseline analysis.**

*df*: degrees of freedom. CFI: comparative fit index; TLI: Tucker–Lewis index; RMSEA: root mean square error of approximation; SRMR: standardized root mean squared residual.

^†^Meets the requirement for good model fit.

**Supplementary Table 5. Results from sex-specific analysis showing standardized regression coefficients and model evaluation statistics for cross-lagged panel models.**

|  | **Women**  **N=9048** | **Men**  **N=1870** |
| --- | --- | --- |
| **Baseline** |  |  |
| Mental health **🡪** Depression | 0.90* | 0.87* |
| Mental health **🡪** Anxiety | 0.76*** | 0.82*** |
| Social network **🡪** Family closeness | 0.66* | 0.70* |
| Social network **🡪** Warmth from others | 0.85*** | 0.82*** |
| Social network **🡪** Societal cohesion | 0.52*** | 0.51*** |
| **One-year follow-up** |  |  |
| Mental health **🡪** Depression | 0.92* | 0.90* |
| Mental health **🡪** Anxiety | 0.81*** | 0.82*** |
| Social network **🡪** Family closeness | 0.69* | 0.69* |
| Social network **🡪** Warmth from others | 0.85*** | 0.85*** |
| Social network **🡪** Societal cohesion | 0.55*** | 0.52*** |
| **Cross-lagged paths** |  |  |
| Mental health (one year) **🡪** Mental health (baseline) | 0.56*** | 0.66*** |
| Social network (one year) **🡪** Social network (baseline) | 0.49*** | 0.46*** |
| **Model Fit** |  |  |
| $\boldsymbol{\chi}^{\boldsymbol{2}}$/*df* | 2277.23/31*** | 488.59/31*** |
| CFI | 0.930 | 0.932 |
| TLI | 0.898 | 0.901 |
| RMSEA | 0.089 | 0.089 |
| SRMR | 0.037^†^ | 0.046^†^ |

*** Coefficients on paths from latent variables to first observed endogenous variables set to normalization constraint of 1 and therefore do not have a p value.**

***** p < 0.001**

**Model 3 was used for baseline analysis.**

*df*: degrees of freedom. CFI: comparative fit index; TLI: Tucker–Lewis index; RMSEA: root mean square error of approximation; SRMR: standardized root mean squared residual.

^†^Meets the requirement for good model fit.

**Supplementary Table 6. Results from age-specific analysis showing standardized regression coefficients and model evaluation statistics for cross-sectional structural equation models (18-39: N=2398; 40-59: N=4356; 60+: N=4164).**

|  | **Baseline** | | | **Six-month follow-up** | | | **One-year follow-up** | | |
| --- | --- | --- | --- | --- | --- | --- | --- | --- | --- |
|  | **18-39** | **40-59** | **60+** | **18-39** | **40-59** | **60+** | **18-39** | **40-59** | **60+** |
| Mental health **🡪** Depression | 1.12* | 1.12* | 1.21* | 0.98* | 0.98* | 1.03* | 0.97* | 0.97* | 0.97* |
| Mental health **🡪** Anxiety | 0.62*** | 0.62*** | 0.57*** | 0.77*** | 0.77*** | 0.72*** | 0.77*** | 0.77*** | 0.76*** |
| Social network **🡪** Family closeness | 0.64* | 0.64* | 0.67* | 0.62* | 0.62* | 0.61* | 0.66* | 0.66* | 0.70* |
| Social network **🡪** Warmth from others | 0.90*** | 0.90*** | 0.88*** | 0.91*** | 0.91*** | 0.91*** | 0.89*** | 0.89*** | 0.89*** |
| Social network **🡪** Societal cohesion | 0.51*** | 0.51*** | 0.47*** | 0.51*** | 0.51*** | 0.47*** | 0.53*** | 0.53*** | 0.51*** |
| Mental health **🡪** Social network | -0.15*** | -0.15*** | -0.10*** | -0.21*** | -0.21*** | -0.17*** | -0.22*** | -0.22*** | -0.20*** |
|  |  |  |  |  |  |  |  |  |  |
| **Model Fit** |  |  |  |  |  |  |  |  |  |
| $\boldsymbol{\chi}^{\boldsymbol{2}}$/*df* | 47.99/4*** | 47.99/4*** | 15.03/4** | 52.95/4*** | 52.95/4*** | 18.55/4*** | 52.70/4*** | 52.70/4*** | 23.1/4*** |
| CFI | 0.997^†^ | 0.997^†^ | 0.998^†^ | 0.997^†^ | 0.997^†^ | 0.998^†^ | 0.997^†^ | 0.997^†^ | 0.997^†^ |
| TLI | 0.992^†^ | 0.992^†^ | 0.995^†^ | 0.993^†^ | 0.993^†^ | 0.994^†^ | 0.993^†^ | 0.993^†^ | 0.993^†^ |
| RMSEA | 0.032^†^ | 0.032^†^ | 0.026^†^ | 0.033^†^ | 0.033^†^ | 0.030^†^ | 0.033^†^ | 0.033^†^ | 0.034^†^ |
| SRMR | 0.017^†^ | 0.017^†^ | 0.016^†^ | 0.018^†^ | 0.018^†^ | 0.018^†^ | 0.015^†^ | 0.015^†^ | 0.016^†^ |

*** Coefficients on paths from latent variables to first observed endogenous variables set to normalization constraint of 1 and therefore do not have a p value.**

**** p < 0.01**

***** p < 0.001**

**Model 3 was used for baseline analysis.**

*df*: degrees of freedom. CFI: comparative fit index; TLI: Tucker–Lewis index; RMSEA: root mean square error of approximation; SRMR: standardized root mean squared residual.

^†^Meets the requirement for good model fit.

**Supplementary Table 7. Results from age-specific analysis showing standardized regression coefficients and model evaluation statistics for cross-lagged panel models.**

|  | **18-39**  **N=2398** | **40-59**  **N=4356** | **60+**  **N=4164** |
| --- | --- | --- | --- |
| **Baseline** |  |  |  |
| Mental health **🡪** Depression | 0.90* | 0.90* | 0.88* |
| Mental health **🡪** Anxiety | 0.77*** | 0.77*** | 0.77*** |
| Social network **🡪** Family closeness | 0.67* | 0.67* | 0.71* |
| Social network **🡪** Warmth from others | 0.85*** | 0.85*** | 0.81*** |
| Social network **🡪** Societal cohesion | 0.52*** | 0.52*** | 0.48*** |
| **One year** |  |  |  |
| Mental health **🡪** Depression | 0.92* | 0.92* | 0.92* |
| Mental health **🡪** Anxiety | 0.82*** | 0.82*** | 0.80*** |
| Social network **🡪** Family closeness | 0.69* | 0.69* | 0.74* |
| Social network **🡪** Warmth from others | 0.85*** | 0.85*** | 0.84*** |
| Social network **🡪** Societal cohesion | 0.54*** | 0.54*** | 0.51*** |
| **Cross-lagged paths** |  |  |  |
| Mental health (one year) **🡪** Mental health (baseline) | 0.58*** | 0.58*** | 0.58*** |
| Social network (one year) **🡪** Social network (baseline) | 0.48*** | 0.48*** | 0.50*** |
| **Model Fit** |  |  |  |
| $\boldsymbol{\chi}^{\boldsymbol{2}}$/*df* | 2713.71/31*** | 2713.71/31*** | 1023.46/31*** |
| CFI | 0.931 | 0.931 | 0.931 |
| TLI | 0.899 | 0.899 | 0.900 |
| RMSEA | 0.089 | 0.089 | 0.088 |
| SRMR | 0.038^†^ | 0.038^†^ | 0.040^†^ |

*** Coefficients on paths from latent variables to first observed endogenous variables set to normalization constraint of 1 and therefore do not have a p value.**

***** p < 0.001**

**Model 3 was used for baseline analysis.**

*df*: degrees of freedom. CFI: comparative fit index; TLI: Tucker–Lewis index; RMSEA: root mean square error of approximation; SRMR: standardized root mean squared residual.

^†^Meets the requirement for good model fit.

**Supplementary Material: R code for SEM analysis**

# Load required packages

library(lavaan)

library(semPlot)

************************************************************************************************

Cross-sectional models

************************************************************************************************

# Unadjusted cross-sectional SEM at baseline

cmodel.0 <- '

# Measurement model

mh.0 =~ dep0 + anx0

sn.0 =~ ss4.0 + ss5.0 + ss6.0

# Regression

mh.0 ~ sn.0

'

fit1 <- sem(cmodel.0, data = df.unadj)

summary(fit1, fit.measures=TRUE, standardized = TRUE)

standardizedSolution(fit1)

************************************************************************************************

# Unadjusted cross-sectional SEM at 6 months

cmodel.6 <- '

# Measurement model

mh.6 =~ dep6 + anx6

sn.6 =~ ss4.6 + ss5.6 + ss6.6

# Regression

mh.6 ~ sn.6

'

fit2 <- sem(cmodel.6, data = df.unadj)

summary(fit2, fit.measures=TRUE, standardized = TRUE)

standardizedSolution(fit2)

************************************************************************************************

# Unadjusted cross-sectional SEM at 12 months

cmodel.12 <- '

# Measurement model

mh.12 =~ dep12 + anx12

sn.12 =~ ss4.12 + ss5.12 + ss6.12

# Regression

mh.12 ~ sn.12

'

fit3 <- sem(cmodel.12, data = df.unadj)

summary(fit3, fit.measures=TRUE, standardized = TRUE)

standardizedSolution(fit3)

************************************************************************************************

# Baseline Model 1 – adjusted for age and sex

model.adjm1 <- '

# Measurement model

mh.m1 =~ dep.m1 + anx.m1

sn.m1 =~ ss4.m1 + ss5.m1 + ss6.m1

# Regression

mh.m1 ~ sn.m1

'

fit.adjm1 <- sem(model.adjm1, data = df.adj)

summary(fit.adjm1, fit.measures=TRUE, standardized = TRUE)

standardizedSolution(fit.adjm1)

************************************************************************************************

# Baseline Model 2 – adjusted additionally for previous psychiatric diagnosis, physical comorbidities, BMI, smoking, CAGE score, physical activity, recruitment type

model.adjm2 <- '

# Measurement model

mh.m2 =~ dep.m2 + anx.m2

sn.m2 =~ ss4.m2 + ss5.m2 + ss6.m2

# Regression

mh.m2 ~ sn.m2

'

fit.adjm2 <- sem(model.adjm2, data = df.adj)

summary(fit.adjm2, fit.measures=TRUE, standardized = TRUE)

standardizedSolution(fit.adjm2)

************************************************************************************************

# Baseline Model 3 – adjusted additionally for previous COVID-19 infection, COVID-19-related worries, economic difficulties, employment status, response period

model.adjm3 <- '

# Measurement model

mh.m3 =~ dep.m3 + anx.m3

sn.m3 =~ ss4.m3 + ss5.m3 + ss6.m3

# Regression

mh.m3 ~ sn.m3

'

fit.adjm3 <- sem(model.adjm3, data = df.adj)

summary(fit.adjm3, fit.measures=TRUE, standardized = TRUE)

standardizedSolution(fit.adjm3)

************************************************************************************************

# 6 months "Model 4" – fully adjusted

model.adjm4 <- '

# Measurement model

mh.m4 =~ dep.m4 + anx.m4

sn.m4 =~ ss4.m4 + ss5.m4 + ss6.m4

# Regression

mh.m4 ~ sn.m4

'

fit.adjm4 <- sem(model.adjm4, data = df.adj)

summary(fit.adjm4, fit.measures=TRUE, standardized = TRUE)

standardizedSolution(fit.adjm4)

************************************************************************************************

#12 months "Model 5" – fully adjusted

model.adjm5 <- '

# Measurement model

mh.m5 =~ dep.m5 + anx.m5

sn.m5 =~ ss4.m5 + ss5.m5 + ss6.m5

# Regression

mh.m5 ~ sn.m5

'

fit.adjm5 <- sem(model.adjm5, data = df.adj)

summary(fit.adjm5, fit.measures=TRUE, standardized = TRUE)

standardizedSolution(fit.adjm5)

************************************************************************************************

Cross-lagged panel models

************************************************************************************************

# Unadjusted CLPM

xlag.unadj <- '

# Measurement models

# Baseline

mh.0 =~ dep0 + anx0

sn.0 =~ ss4.0 + ss5.0 + ss6.0

# 12 months

mh.12 =~ dep12 + anx12

sn.12 =~ ss4.12 + ss5.12 + ss6.12

# Latent autoregressive paths

mh.12 ~ c_mh.0 * mh.0

sn.12 ~ c_sn.0 * sn.0

# Cross-lagged paths

mh.12 ~ b_sn.0 * sn.0

sn.12 ~ b_mh.0 * mh.0

# Residual covariances

mh.0 ~~ sn.0

mh.12 ~~ sn.12

'

fit.xunadj <- sem(xlag.unadj, data = df.unadj)

summary(fit.xunadj, fit.measures=TRUE, standardized = TRUE)

standardizedSolution(fit.xunadj)

************************************************************************************************

# Adjusted model 1: adjusted for age and sex

xlag.m1 <- '

# Measurement models

# Baseline

mh.m1 =~ dep.m1 + anx.m1

sn.m1 =~ ss4.m1 + ss5.m1 + ss6.m1

# 12 months

mh.m5 =~ dep.m5 + anx.m5

sn.m5 =~ ss4.m5 + ss5.m5 + ss6.m5

# Latent autoregressive paths

mh.m5 ~ c_mh.m1 * mh.m1

sn.m5 ~ c_sn.m1 * sn.m1

# Residual covariances

mh.m1 ~~ sn.m1

mh.m5 ~~ sn.m5

'

fit.xlagm1 <- sem(xlag.m1, data = df.adj)

summary(fit.xlagm1, fit.measures=TRUE, standardized = TRUE)

standardizedSolution(fit.xlagm1)

************************************************************************************************

# Adjusted model 2: adjusted additionally for previous psychiatric diagnosis, physical comorbidities, BMI, smoking, CAGE score, physical activity, and recruitment type

xlag.m2 <- '

# Measurement models

# Baseline

mh.m2 =~ dep.m2 + anx.m2

sn.m2 =~ ss4.m2 + ss5.m2 + ss6.m2

# 12 months

mh.m5 =~ dep.m5 + anx.m5

sn.m5 =~ ss4.m5 + ss5.m5 + ss6.m5

# Latent autoregressive paths

mh.m5 ~ c_mh.m2 * mh.m2

sn.m5 ~ c_sn.m2 * sn.m2

# Residual covariances

mh.m2 ~~ sn.m2

mh.m5 ~~ sn.m5

'

fit.xlagm2 <- sem(xlag.m2, data = df.adj)

summary(fit.xlagm2, fit.measures=TRUE, standardized = TRUE)

standardizedSolution(fit.xlagm2)

************************************************************************************************

# Adjusted model 3: adjusted additionally for previous COVID-19 infection, COVID-19-related worries, economic difficulties, employment status, and response period

xlag.m3 <- '

# Measurement models

# Baseline

mh.m3 =~ dep.m3 + anx.m3

sn.m3 =~ ss4.m3 + ss5.m3 + ss6.m3

# 12 months

mh.m5 =~ dep.m5 + anx.m5

sn.m5 =~ ss4.m5 + ss5.m5 + ss6.m5

# Latent autoregressive paths

mh.m5 ~ c_mh.m3 * mh.m3

sn.m5 ~ c_sn.m3 * sn.m3

# Cross-lagged paths

mh.m5 ~ b_sn.m3 * sn.m3

sn.m5 ~ b_mh.m3 * mh.m3

# Residual covariances

mh.m3 ~~ sn.m3

mh.m5 ~~ sn.m5

'

fit.xlagm3 <- sem(xlag.m3, data = df.adj)

summary(fit.xlagm3, fit.measures=TRUE, standardized = TRUE)

standardizedSolution(fit.xlagm3)
